# Neural foundation of the diathesis-stress model: longitudinal gray matter volume changes in response to stressful life events in major depressive disorder and healthy controls

**DOI:** 10.1038/s41380-024-02526-4

**Published:** 2024-03-29

**Authors:** Florian Thomas-Odenthal, Kai Ringwald, Lea Teutenberg, Frederike Stein, Nina Alexander, Linda M. Bonnekoh, Katharina Brosch, Katharina Dohm, Kira Flinkenflügel, Dominik Grotegerd, Tim Hahn, Andreas Jansen, Elisabeth J. Leehr, Susanne Meinert, Julia-Katharina Pfarr, Harald Renz, Navid Schürmeyer, Thomas Stief, Benjamin Straube, Katharina Thiel, Paula Usemann, Alexandra Winter, Axel Krug, Igor Nenadić, Udo Dannlowski, Tilo Kircher

**Affiliations:** 1 Department of Psychiatry and Psychotherapy, University of Marburg, Marburg, Germany; 2 Center for Mind, Brain and Behavior (CMBB), Universities of Marburg and Gießen, Marburg, Germany; 3 Institute for Translational Psychiatry, University of Münster, Münster, Germany; 4 Department of Child and Adolescent Psychiatry, University Hospital Münster, Münster, Germany; 5 Core-Facility BrainImaging, Faculty of Medicine, University of Marburg, Marburg, Germany; 6 Institute for Translational Neuroscience, University of Münster, Münster, Germany; 7 Institute of Laboratory Medicine and Pathobiochemistry, Molecular Diagnostics, University of Marburg, Marburg, Germany; 8 Department of Psychiatry and Psychotherapy, University Hospital of Bonn, Bonn, Germany

**Keywords:** Depression, Neuroscience

## Abstract

Recurrences of depressive episodes in major depressive disorder (MDD) can be explained by the diathesis-stress model, suggesting that stressful life events (SLEs) can trigger MDD episodes in individuals with pre-existing vulnerabilities. However, the longitudinal neurobiological impact of SLEs on gray matter volume (GMV) in MDD and its interaction with early-life adversity remains unresolved. In 754 participants aged 18–65 years (362 MDD patients; 392 healthy controls; HCs), we assessed longitudinal associations between SLEs (Life Events Questionnaire) and whole-brain GMV changes (3 Tesla MRI) during a 2-year interval, using voxel-based morphometry in SPM12/CAT12. We also explored the potential moderating role of childhood maltreatment (Childhood Trauma Questionnaire) on these associations. Over the 2-year interval, HCs demonstrated significant GMV reductions in the middle frontal, precentral, and postcentral gyri in response to higher levels of SLEs, while MDD patients showed no such GMV changes. Childhood maltreatment did not moderate these associations in either group. However, MDD patients who had at least one depressive episode during the 2-year interval, compared to those who did not, or HCs, showed GMV increases in the middle frontal, precentral, and postcentral gyri associated with an increase in SLEs and childhood maltreatment. Our findings indicate distinct GMV changes in response to SLEs between MDD patients and HCs. GMV decreases in HCs may represent adaptive responses to stress, whereas GMV increases in MDD patients with both childhood maltreatment and a depressive episode during the 2-year interval may indicate maladaptive changes, suggesting a neural foundation for the diathesis-stress model in MDD recurrences.

## Introduction

Stressful life events (SLEs), such as personal loss or moving to a new place, are a significant predictor for the onset and recurrence of major depressive disorder (MDD) [1]. SLEs often trigger MDD episodes in individuals predisposed to MDD, a relationship conceptualized decades ago by the diathesis-stress model [2]. This model suggests that individuals with vulnerability factors, such as genetic predispositions [3] and/or early environmental influences like childhood maltreatment (CM) [4, 5], are more likely to develop MDD when exposed to SLEs in adulthood [2]. SLEs can also trigger recurrent MDD episodes [6]. However, not all individuals exposed to these stressors develop MDD or experience MDD recurrences [1], indicating individual differences in neurobiological responses to stress.

Cross-sectional structural magnetic resonance imaging (MRI) studies have connected recent SLEs in adulthood with gray matter volume (GMV) alterations of the insula, anterior cingulate, and medial prefrontal and medial orbitofrontal cortices in healthy subjects [7–10]. MDD patients showed a different pattern with fewer GMV alterations in the medial orbitofrontal cortex than healthy controls (HCs) [10].

In another line of cross-sectional MRI studies, HCs and MDD patients with self-reported CM – the best documented early environmental risk factor – showed smaller GMV of the dorsolateral prefrontal and anterior cingulate cortices, supplementary motor area, postcentral gyrus, amygdala, and hippocampus compared to those without such history [11–13]. CM was also found to moderate the relationship between SLEs on GMV in MDD patients, but not in HCs, hinting at a brain structural perspective on the diathesis-stress model in MDD [10].

Despite these findings, our understanding of the impact of SLEs on brain structure is still limited due to a lack of longitudinal studies. It is unclear whether the previously observed brain structural correlates are a consequence of stress or indicative of a predisposition to experience more SLEs. To date, two longitudinal studies showed GMV reductions in response to recent SLEs in HCs of the anterior cingulate, hippocampus, parahippocampal gyrus, and medial prefrontal gyrus [14, 15]. However, the nature of these reductions, representing adaptive or maladaptive responses to stress, remains unclear due to a lack of cohort studies of MDD patients. By comparing longitudinal brain structural changes of MDD patients with HCs, we could clarify whether these changes resulted from MDD development. Addressing these gaps would enable the translation of the long-established biopsychosocial diathesis-stress model into a neurobiological framework to better understand the brain structural underpinnings of MDD and its recurrent episodes.

Therefore, we investigated, for the first time, the relationship between SLEs and GMV changes in a large group of MDD patients compared to HCs during a 2-year investigational interval, within the context of the diathesis-stress model. We hypothesized that HCs would show greater GMV reductions in response to SLEs compared to MDD patients during the 2-year interval, consistent with previous cross-sectional studies. Furthermore, we hypothesized that CM would moderate the relationship between SLEs and GMV changes only in MDD patients, but not HCs when directly compared. We further explored this relationship in MDD patients with more severe forms of depression and in particular those who had at least one depressive episode during the 2-year interval, considering their increased vulnerability to SLEs. Lastly, we explored the role of C-reactive protein (CRP) – a systematic marker of inflammation – as a potential moderator of the relationship between SLEs and GMV changes in MDD patients and HCs, as elevated CRP levels have been associated with stress and the onset and recurrence of depression as well as GMV changes (for an in-depth rationale, see Supplementary 1).

## Subjects and methods

### Participants

754 participants (*n* = 392 HC; *n* = 362 MDD) were included in this analysis from the ongoing Marburg–Münster Affective Disorder Cohort Study (MACS) [16]. MACS is part of the FOR2107, a consortium that investigates the neurobiology of major psychiatric disorders. All relevant data pertaining to our research question were used for this analysis to detect clinically significant effects. Part of this study’s data were previously analyzed in a cross-sectional design on the effects of SLEs on GMV in MDD patients and HCs [10]. All participants underwent T1-weighted MRI scans and clinical assessments by trained staff at both baseline (T1) and follow-up (T2) time points, with the follow-up (T2) assessment occurring approximately 2 years after the baseline (T1) assessment (mean = 2.22 years, SD = 0.31, range: 1.9–4.3 years). Both assessments took place at the University of Marburg and the University of Münster in Germany. Inclusion criteria required participants to be aged 18–65 years old at baseline (T1) time point. Exclusion criteria were a history of neurological or general medical conditions, current substance dependence, and verbal intelligence quotient (IQ) ≤ 80. Further exclusion criteria for the HC group were current or past mental disorders per Structured Clinical Interview for DSM-IV-TR (SCID-I) [17], and lifetime intake of psychotropic medication (for details, see elsewhere) [16]. Ethical approval was obtained from the ethics committees of the medical faculties at the University of Marburg (AZ: 07/14) and the University of Münster (AZ: 2014-422-b-S) following the Declaration of Helsinki. Participants provided written informed consent and received financial compensation.

#### Assessment of clinical-psychosocial variables

In a semi-structured interview, clinical variables were assessed, such as course of illness (number and duration of depressive episodes, number and duration of hospitalization), current remission status (partially or fully remitted, according to SCID-I criteria), psychopathology (17-item Hamilton Depression Rating Scale; HAM-D, state anxiety subscale of the State-Trait Anxiety Inventory; STAI-S) [18, 19], social functioning (Global Assessment of Functioning; GAF) [20], and current medication intake. Other rater-based and self-report scales included familial risk (asking if a first-degree relative had been treated for MDD, bipolar disorder, schizophrenia, or schizoaffective disorder, considered collectively), perceived stress (Perceived Stress Scale questionnaire; PSS) [21], neuroticism (NEO Five-Factor Inventory questionnaire; NEO-FFI) [22], resilience (25-item Resilience Scale; RS-25) [23], social support (Fragebogen zur Sozialen Unterstützung; FSozU) [24], and attachment style (Relationship Scales Questionnaire; RSQ) [25].

#### Assessment of childhood maltreatment

CM was evaluated using the Childhood Trauma Questionnaire (CTQ) [26] on the domains of emotional abuse, physical abuse, sexual abuse, emotional neglect, and physical neglect. The CTQ measures to what extent these events applied to their childhood using a five-point scale ranging from “not at all” to “very often” (scored 0–4). The cumulative experiences of CM were represented by the CTQ sum score.

#### Assessment of stressful life events

We used the “adaptation” framework by Cohen et al. [1] to assess recent SLEs occurring between baseline (T1) and follow-up (T2) time points. SLEs were defined as any event, positive or negative, that significantly impacted a person’s life and required adaptation. The Life Events Questionnaire (LEQ) [27] assessed the cumulative impact of SLEs during the 2-year interval (T2-T1), including 79 items covering domains such as health, work, finance, law, and personal and social life, amongst others. Participants rated the impact of these events since baseline (T1) time point on a 0–3 scale from “no effect” to “great effect”. From this, three scores were calculated: the negative events score (sum of all negatively perceived events), the positive events score (sum of all positively perceived events), and the total events score (sum of both events). We used the LEQ total events score for all analyses, capturing the cumulative impact of SLEs during the 2-year interval (T2-T1), collected at follow-up (T2) time point. For descriptive statistics refer to Table 1.

**Table 1:** Descriptive statistics of study participants at baseline (T1) and follow-up (T2) time points.

	Baseline (T1)			Follow-up (T2)		
	HC (*n* = 392)	MDD (*n* = 362)	*P*	HC (*n* = 392)	MDD (*n* = 362)	*P*
Age	34.97 (13.28)	35.47 (12.90)	0.453	37.18 (13.27)**	37.73 (12.92)**	0.413
Sex, *n*	F = 238, M = 154	F = 230, M = 132	0.425	-	-	-
BMI	24.21 (4.22)	25.65 (17.01)	0.004	24.68 (4.10)	26.49 (5.78)	<0.001
TIV	1533.63 (139.48)	1528.65 (148.99)	0.405	1531.18 (139.80)**	1527.15 (148.33)*	0.437
HAM-D	1.24 (1.84)	8.07 (6.24)	<0.001	1.05 (1.68)*	5.66 (5.45)**	<0.001
GAF	91.65 (7.33)	65.67 (13.66)	<0.001	90.40 (7.84)*	71.78 (13.93)**	<0.001
STAIS	33.76 (8.09)	49.15 (12.24)	<0.001	31.05 (7.84)*	41.51 (12.23)**	<0.001
FSozU/SSQ	4.54 (0.50)	3.82 (0.85)	<0.001	4.58 (0.48)*	4.02 (0.80)**	<0.001
PSS	15.86 (6.99)	28.67 (9.57)	<0.001	16.30 (6.61)	23.75 (8.90)**	<0.001
RS25	142.51 (17.48)	112.46 (24.94)	<0.001	142.51 (16.57)	117.75 (23.32)**	<0.001
RSQ secure, *n*	264 (67.34%)	89 (24.58%)	<0.001	-	-	-
NEOFFI neuroticism	14.82 (7.20)	28.09 (9.32)	<0.001	-	-	-
First-degree relative with MDD, BD, SCZ, or SZA, *n* (%)	86 (21.93%)	130 (35.91%)	<0.001	-	-	-
hsCRP, mg/L	1.95 (3.73)	2.63 (5.01)	0.874^a^	-	-	-
Smoking status, *n* (%)	36 (9.18%)	73 (20.16%)	<0.001	-	-	-
NSAID, *n* (%)	0 (0%)	9 (2.48%)	0.002	-	-	-
Remission status	-	*a* = 145, *r* = 216	-	-	*a* = 54, *r* = 308**	-
Antipsychotics, *n* (%)	-	60 (16.57%)	-	-	40 (11.04%)*	-
Antidepressants, *n* (%)	-	215 (59.39%)	-	-	160 (44.19%)**	-
Lithium, *n* (%)	-	6 (1.65%)	-	-	11 (3.03%)	-
Number of reported SLEs between T1 and T2	-	-	-	11.06 (6.18)	14.01 (7.03)	<0.001
LEQ total events score	-	-	-	20.35 (13.64)	29.36 (17.15)	<0.001
LEQ negative events score	-	-	-	5.57 (6.11)	12.21 (10.97)	<0.001
LEQ positive events score	-	-	-	14.78 (10.45)	17.15 (12.25)	0.017
CTQ	-	-	-	31.51 (7.05)	43.91 (15.04)	<0.001
Interscan interval, days	-	-	-	808.00 (112.03)	810.86 (116.41)	0.247
At least one depressive episode between T1 and T2, *n* (%)	-	-	-	-	162 (44.75%)	-
Number of depressive episodes between T1 and T2	-	-	-	-	0.70 (0.95)	-
Duration of depressive episodes between T1 and T2 [months]	-	-	-	-	4.92 (6.62)	-
Number of hospitalizations between T1 and T2	-	-	-	-	0.32 (0.77)	-
Duration of hospitalization between T1 and T2 [months]	-	-	-	-	0.98 (2.66)	-

All values are given as mean (*SD*) unless otherwise specified.

*BD* bipolar disorder, *BMI* body mass index, *CTQ* childhood trauma questionnaire, *FSozU/SSQ* social support questionnaire, *GAF* Global Assessment of Functioning, *F* female, *M* male, *HAM-D* Hamilton Depression Rating Scale, *HC* healthy control, *hsCRP* high-sensitivity C-reactive protein, *LEQ* Life Events Questionnaire, *MDD* major depressive disorder, *NEOFFI* NEO Five-Factor Inventory questionnaire, *NSAID* nonsteroidal anti-inflammatory drugs, *PSS* Perceived Stress Scale questionnaire, *RSQ* Relationship Scales Questionnaire, *RS25* 25-item Resilience Scale, *SCZ* schizophrenia, *SLEs* stressful life events, *STAI-S* State-Trait Anxiety Inventory, *SZA* schizoaffective disorder, *a* acute, *r* partially or fully remitted (according to SCID-I/DSM-IV-TR), *n* number of participants.

*P*-values stem from the non-parametric Kruskal–Wallis test for between-group comparisons, or the Wilcoxon signed-rank test for within-group comparisons

*Significant within-group differences between baseline and follow-up at *p* < 0.05;

**Significant within-group differences between baseline and follow-up at *p* < 0.001.

^a^hs-CRP data was only available for 509 participants (HCs: *n* = 265; MDD: *n* = 244).

### MRI acquisition and pre-processing

#### MRI acquisition

MRI data were acquired at both baseline (T1) and follow-up (T2) time points using 3 Tesla MRI scanners (Siemens, Erlangen, Germany) with standardized pulse sequence parameters and extensive quality assurance protocols (for an overview, see elsewhere) [28]. T1-weighted images were obtained using a three-dimensional MP-RAGE sequence with a slice thickness of 1 mm (voxel size of 1 × 1 × 1 mm), and a field of view of 256 mm. In Marburg, a Tim Trio scanner was used with a 12-channel head matrix Rx-coil with the following parameters: TR = 1.9 s, TE = 2.26 ms, TI = 900 ms, flip angle = 9°. In Münster, a Prisma Fit was used with a 20-channel head matrix Rx-coil with the following parameters: TR = 2.13 s, TE = 2.28 ms, TI = 900 ms, flip angle = 8°.

#### MRI pre-processing

The T1-weighted scans were pre-processed using the longitudinal pre-processing pipeline of the CAT12 toolbox (Computational Anatomy toolbox, v1720, Structural Brain Mapping Group, Jena, Germany) as implemented in SPM12 (Statistical Parametric Mapping, Institute of Neurology, London, UK) running on MATLAB (version R2017a, The MathWorks, Natick, Massachusetts, USA). Default parameter settings were used including realignment, bias correction, tissue classification, and spatial normalization using the Geodesic Shooting template. Images were segmented into gray matter, white matter, and cerebrospinal fluid, and smoothed with an 8 mm full width at half maximum (FWHM) Gaussian kernel. Data were normalized to Montreal Neurological Institute (MNI) space, and total intracranial volume (TIV) was calculated. GMV was computed by modulating gray matter tissue probability maps with the non-linear deformation fields from the normalization procedure. Individual quality control measures were performed, including visual inspection and identification of outliers using the check homogeneity function in CAT12 to ensure all data were free from artifacts and abnormal brain structure. Between the baseline (T1) and follow-up (T2) time points, the body coil and gradient coil were exchanged at the Marburg site in June 2016 and August 2018, resulting in two dummy-coded variables (yes/no) for each coil change to account for potential differences in scanner settings occurring within subject assessments.

### Statistical analyses

#### Whole-brain analyses for longitudinal data

Our goal was to examine how SLEs, quantified by the LEQ total events score, are associated with changes in GMV over time between HCs and MDD patients. For this purpose, we performed a 2 × 2 repeated measures Analysis of Covariance (ANCOVA) using the flexible factorial design in SPM12/CAT12. Subject, scanning time point (baseline [T1] and follow-up [T2]), and group were included as main factors. The interaction between LEQ total events score and group was included as a covariate of interest. Age, sex, and interscan interval (time in days between baseline [T1] and follow-up [T2] scans) were included as covariates of no interest at follow-up (T2) time point and set to zero at baseline (T1), as they might affect stress responses and brain structural changes over time [29, 30]. Furthermore, we included two dummy-coded variables for body coil and gradient coil changes in the model; TIV was not included as a covariate because each subject acted as their control. A threshold of 0.1 was applied to the absolute gray matter values. Cluster-level significance was set at *p* < 0.05 (one-tailed) FWE corrected for multiple comparisons. The eigenvariate function in SPM was used to extract the significant cluster values (the weighted means of intensity values) for visualization and further analyses, such as statistical model building or control analyses in Jamovi software [31]. To understand the potential differential impact of positive and negative life events on GMV changes, we tested for differences in correlations between LEQ positive events score and GMV change, and LEQ negative events score and GMV change, using Steiger’s Z [32].

#### Moderation analyses for longitudinal data

To investigate potential interactions between SLEs and CM in influencing GMV changes, multiple linear regression analysis was employed using three-way interactions. Specifically, we assessed the interaction between LEQ total events score, CTQ sum score, and group (MDD vs HC) on the change in extracted values of significant clusters (GMV follow-up [T2] – GMV baseline [T1]). Age and sex at follow-up (T2), interscan interval, and the scanner variables were again included in the model as covariates of no interest. All main effects and two-way interactions of LEQ total events score, CTQ sum score, and group variable were also accounted for. We further explored the potential moderating role of high-sensitivity C-reactive protein (hsCRP) at baseline (T1) on the relationship between SLEs and GMV changes (for details, see Supplementary Methods). Data were visually inspected for normality and homoscedasticity using residual- and Q-Q-plots on the fitted and standardized residuals, respectively. Significance level was set at *p* < 0.05 (one-tailed), Bonferroni corrected for multiple comparisons.

#### Moderation analyses for longitudinal data in MDD patients

We further explored whether MDD patients with a more severe form of depression, and in particular those with at least one depressive episode during the 2-year interval were more susceptible to the effects of SLEs and CM on GMV changes. For this exploration, we first conducted a whole-brain analysis of SLEs on GMV change in MDD patients with and without an episode and HCs using a 3 × 2 repeated measures ANCOVA flexible-factorial design in SPM12/CAT12. We retained the scanner settings and covariates from our confirmatory whole-brain analysis as described above. Subsequent moderation analyses were employed on the extracted weighted means of intensity values from identified clusters. Severity was operationalized by number of hospitalizations, duration of hospitalization, number of depressive episodes, duration of depression during the 2-year interval (T2-T1), and remission status at follow-up (T2) time point. To create time-adjusted measures, we divided variables capturing events during the 2-year interval (T2-T1), like the number or duration of depressive episodes, by the duration of the interscan interval. Significance level was set at *p* < 0.05 (two-tailed), Bonferroni corrected for multiple comparisons.

#### Control analyses for longitudinal data

To explore relationships between brain structure and other potential influencing factors, as well as their potential interactions with SLEs on GMV change, we performed additional control analyses. These were run using ANCOVA, Pearson or Spearman’s rho correlations, depending on the data distribution. These variables included clinical factors (duration of hospitalization during the interval, number of depressive episodes during the 2-year interval [T2-T1], remission status and depression severity at follow-up [T2] time point), familial risk, psychological factors (state anxiety, perceived stress, neuroticism, social support, resilience, and attachment style), and other factors (global functioning and medication intake) assessed at follow-up (T2) time point. Significance level was set at *p* < 0.05 (two-tailed), Bonferroni corrected for multiple comparisons.

#### Whole-brain analyses for cross-sectional data

To explore predictive or retrospective cross-sectional associations between baseline (T1) and follow-up (T2) GMV and SLEs during the 2-year interval (T2-T1), respectively, ANCOVAs using the full factorial (between-subjects) design in SPM were run. A baseline (T1) cross-sectional but no longitudinal (T2-T1) association would suggest that GMV alterations might predict the experience of future SLEs instead of SLEs causing GMV changes. The cross-sectional models included the covariates age, sex, TIV, and two dummy-coded variables for body coil and gradient coil differences. Cluster-level significance was set at *p* < 0.05 (two-tailed), FWE corrected for multiple comparisons at a threshold of *k* = 10 voxels.

## Results

### Associations between SLEs and GMV change

#### Whole-brain analysis

**Fig. 1:**
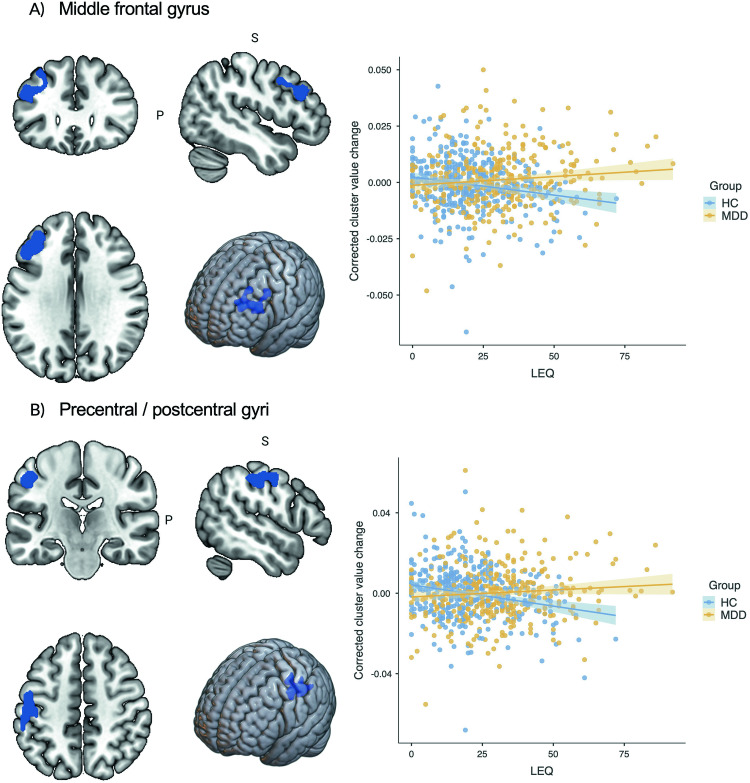
Association between stressful life events (LEQ) and GMV change between MDD patients and HCs during the 2-year interval. Figure shows the relationship between stressful life events (LEQ) and change in corrected cluster values during the 2-year interval in (**A**) the middle frontal gyrus and (**B**) the precentral/postcentral gyri between MDD patients and HCs. HCs experienced larger GMV reductions in these areas the more stressful life events they experienced during the 2-year interval, whereas MDD patients did not show such significant GMV changes with increasing stressful life events.

Repeated measures 2 × 2 ANCOVA in SPM identified two significant clusters upon comparing the effects of SLEs on longitudinal GMV changes during the 2-year interval (T2-T1) between HCs and MDD patients. The first cluster included parts of the left middle frontal gyrus (*k* = 1588 voxels, x/y/z = -46/27/28, *t*_1,745_ = 4.02 FWE cluster-level, Cohen’s *d* = 0.29, *p* = 0.005), and the second cluster the left precentral and postcentral gyri (*k* = 1364 voxels, x/y/z = -52/-22/44, *t*_1,745_ = 4.50 FWE cluster-level, Cohen’s *d* = 0.33, *p* = 0.009; for percentages of cluster distribution, see Supplementary Table S1). Results indicated that HCs had larger GMV reductions in these areas the more SLEs they experienced during the 2-year interval (middle frontal gyrus: *β* = −0.18, *t* = −3.45, *p* < 0.001; postcentral/precentral gyri: *β* = −0.21, *t* = 4.20, *p* < 0.001). MDD patients did not show such significant GMV changes with increasing SLEs (middle frontal gyrus: *β* = 0.10*, t* = 1.77, *p* = 0.077; postcentral/precentral gyri: *β* = 0.07, *t* = 1.21, *p* = 0.227; see Fig. 1). Moreover, there were no significant clusters for MDD patients having larger GMV reductions with increasing SLEs relative to HCs.

Importantly, there were no significant associations between cluster values and clinical and psychosocial variables assessed at follow-up (T2) time point, such as the duration of hospitalization or number of depressive episodes (T2-T1), remission status, familial risk (family history of MDD, bipolar disorder, schizophrenia, or schizoaffective disorder, considered collectively), state anxiety (STAI-S), perceived stress (PSS), neuroticism (NEO-FFI), current depression severity (HAM-D), CM (CTQ), global functioning (GAF), and medication intake (see Supplementary Tables S2 and S3). Furthermore, there were no significant differences in the correlations between LEQ positive events score and GMV change, or LEQ negative events score and GMV change in HCs and MDD patients for both clusters using Steiger’s Z test (see Supplementary Results 1).

### Moderation of CM on the association between SLEs and GMV change

To investigate the diathesis-stress model – the interaction between recent SLEs and CM influencing GMV change (T2-T1) – we employed moderation analyses on the extracted means of intensity values of the identified clusters.

#### Confirmatory linear regression analysis

We found no three-way interaction of LEQ total events score, CTQ sum score, and group on GMV change in both clusters (middle frontal gyrus: *β* = −0.02, *t* = −0.41, *p* = 0.681; precentral/postcentral gyri: *β* = 0.03, *t* = 0.52, *p* = 0.604; for model coefficients, see Supplementary Table S4). This indicates that the influence of SLEs on GMV does not significantly differ between MDD patients and HCs, even when these individuals have experienced higher levels of CM. Moreover, the relationship between SLEs and GMV change among HCs and MDD patients was also not moderated by familial risk (family history of MDD, bipolar disorder, schizophrenia, or schizoaffective disorder, considered collectively), state anxiety (STAI-S), perceived stress (PSS), current depression severity (HAM-D), neuroticism (NEO-FFI), secure attachment style (RSQ), resilience (RS-25), or social support (FsozU), all captured at follow-up (T2) time point (see Supplementary Table S5).

#### Exploratory linear regression analyses

**Fig. 2:**
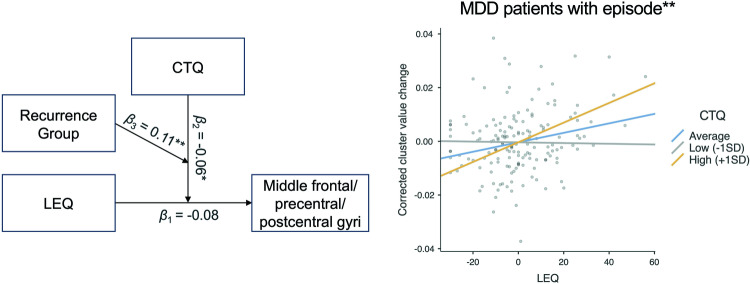
Three-way interaction between stressful life events (LEQ) and childhood maltreatment (CTQ) on GMV change in the middle frontal/precentral/postcentral gyri between MDD patients with and without an episode during the 2-year interval and HCs. Figure illustrates the moderating effect of CTQ on the relationship between LEQ and GMV change in the middle frontal/precentral/postcentral gyri in MDD patients with at least one depressive episode. No significant moderating effect was observed in MDD patients without an episode or HCs during the 2-year interval. The *β*_1_ value represents the simple effect of LEQ on GMV change, *β*_2_ value represents the two-way interaction between LEQ and CTQ on GMV change, and *β*_3_ value represents the three-way interaction between LEQ, CTQ, and recurrence group (MDD patients with an episode during the 2-year interval, MDD patients without an episode, and HCs) on GMV change. *Interactions were statistically significant at *p* < 0.05. **Interactions were statistically significant at *p* < 0.01.

Challenging the diathesis-stress model with exploratory moderation analyses, we found a three-way interaction between LEQ total events score, CTQ sum score, and recurrence group on GMV change in the middle frontal, precentral, and postcentral gyri (*β* = 0.11, *t* = 2.67, *p* = 0.008; for results of whole-brain analyses, see Supplementary Results 2 and Supplementary Fig. S1; for model coefficients of moderation analysis, see Supplementary Table S6). This finding indicates that among MDD patients who experienced an episode during the 2-year interval, higher levels of SLEs were associated with significant GMV increases in the middle frontal, precentral, and postcentral gyri in the context of increased vulnerability due to CM (*β* = 0.23, *t* = 2.83 *p* = 0.005; see Fig. 2), as compared to those patients without an episode (*β* = −0.12, *t* = −1.79, *p* = 0.076) or HCs (*β* = 0.03, *t* = 0.53, *p* = 0.596). This three-way association became even stronger with an increasing number of depressive episodes (middle frontal/precentral/postcentral gyri: *β* = 0.12, *t* = 3.19, *p* = 0.001). Additionally, MDD patients with an episode, as compared to those without or HCs, exhibited a similar GMV increase in response to SLEs in these regions in the context of elevated baseline (T1) hsCRP levels, a pattern not observed in the overall groups of MDD patients and HCs (for detailed results, see Supplementary Results 3, Supplementary Fig. S2, and Supplementary Tables S7 and S8). Detailed descriptive statistics of the recurrence groups are provided in Supplementary Table S9.

Other clinical variables, such as remission status or number of hospitalizations, did not interact with SLEs and CM on GMV changes in MDD patients (LEQ total events score x CTQ sum score x factors; see Supplementary Table S10). Furthermore, the relationship between SLEs and GMV change in MDD patients with vs. without an episode vs. HCs was not moderated by familial risk (family history of MDD, bipolar disorder, schizophrenia, or schizoaffective disorder, considered collectively), remission status (SCID-I), attachment style (RSQ), resilience (PSS), and social support (FsozU) (LEQ total events score x recurrence group x factors; see Supplementary Table S11).

### Predictive and retrospective associations between baseline (T1) or follow-up (T2) GMV and SLEs

To investigate the presence of predictive or retrospective cross-sectional associations in a cluster where no longitudinal association occurs, exploratory whole-brain analyses were run. Six participants were excluded from these analyses due to missing TIV data.

#### Predictive (T1) whole-brain analysis

An ANCOVA indicated a significant interaction between SLEs (LEQ total events score) during the 2-year interval (T2-T1) and group (HC vs. MDD) on baseline (T1) GMV in the precentral and postcentral gyri (*k* = 2 167 voxels, x/y/z = −38/− 32/65, *t*_1,739_ = 4.25 FWE cluster-level, *p* = .002; see Fig. S3). Specifically, as SLEs increased during the 2-year interval (T2-T1), HCs showed larger GMV in this area, whereas MDD patients showed smaller GMV, both at baseline (T1) time point. Furthermore, using a region-of-interest (ROI)-based approach, we found a significant overlap between the predictive cross-sectional (T1) and longitudinal (T2-T1) precentral/postcentral cluster (which was used as ROI) on the interaction between SLEs (T2-T1) and group (*k* = 276 voxels, x/y/z = −52/−10/40, *t*_1,739_ = 3.97, FWE cluster-level, *p* = 0.006; see Supplementary Fig. S4).

#### Retrospective (T2) whole-brain analysis

No significant clusters emerged in the cross-sectional analysis between SLEs during the 2-year interval (T2-T1) and GMV at follow-up (T2) time point.

## Discussion

In this 2-year investigational study, we provide, for the first time, evidence for the longitudinal effects of SLEs on brain structural alterations in a large group of MDD patients and HCs, within the framework of the diathesis-stress model. We found that HCs, but not MDD patients, showed GMV reductions in the middle frontal, precentral, and postcentral gyri, the more they experienced SLEs during the 2-year interval. We found evidence for the diathesis-stress model in MDD patients who experienced at least one depressive episode during the 2-year interval, showing that they exhibited GMV increases in the middle frontal, precentral, and postcentral gyri with increasing SLEs and CM. These associations were independent of medication, clinical characteristics (e.g., duration of depressive episodes, hospitalization duration, remission status, severity of depression or anxiety), psychosocial factors (e.g., social support, resilience, perceived stress, neuroticism, or attachment style), and other variables (e.g., global functioning, familial genetic risk).

Our longitudinal study demonstrates distinct structural neural responses to stress in HCs and MDD patients. HCs had significant GMV reductions (T2-T1) in the middle frontal, precentral, and postcentral gyri in response to SLEs, compared to MDD patients. The middle frontal gyrus is involved in higher-order cognitive functions, such as attention, executive function, and decision-making [33], and is connected to the limbic system, such as the amygdala-hippocampus complex [34–36], crucial for emotional processing [37]. The precentral and postcentral gyri are implicated in sensory, emotional, and motor processes [38, 39]. GMV reductions in these areas in HCs could indicate an adaptive response to stress, where the brain might reallocate neural resources as compensation [13, 40–43]. Alterations of these areas might influence other stress- or resilience-related functional connectivity networks [34–36, 43–45], thereby influencing stress perception, responses, and behaviors [46, 47]. In MDD patients, these adaptive mechanisms may be impaired, resulting in fewer GMV reductions in response to stress [10].

To disentangle the directionality of the observed findings—whether SLEs influence GMV or vice versa—we additionally conducted predictive cross-sectional analyses. Our results suggest that baseline (T1) GMV could predict future SLEs (T2-T1), indicating differential stress vulnerabilities between HCs and MDD patients. HCs with larger, relative to smaller baseline (T1) GMV in the precentral and postcentral gyri were more prone to experiencing higher levels of SLEs in the subsequent 2-year interval (T2-T1). Larger GMV at baseline (T1) time point might therefore indicate an increased inclination to perceive stress or engage in stress-related behaviors, which in turn, could have led to the observed longitudinal GMV reductions (T2-T1), potentially as an adaptive response [35, 38, 46]. Conversely, MDD patients with smaller baseline (T1) GMV in the precentral and postcentral gyri experienced more future SLEs but did not show similar longitudinal GMV reductions. This could imply a plateau in stress-related GMV reductions in MDD patients, potentially due to the disorder itself, or to the experience of previous SLEs [48]. Interestingly, the middle frontal gyrus, implicated in higher cognitive functioning [33], did not show this predictive correlation with SLEs, suggesting that this area might be more directly affected by stress [47].

Within the framework of the diathesis-stress model, which postulates that adult stress interacts with developmental vulnerabilities to influence mental health outcomes [2], we discovered a three-way interaction between adult SLEs, CM, and recurrence group on GMV changes (T2-T1) in the middle frontal, precentral, and postcentral gyri. Specifically, MDD patients with higher levels of CM and at least one depressive episode during the 2-year interval, compared to those without an episode and HCs, showed GMV increases in these areas with increasing SLEs. This finding suggests that MDD patients with a history of CM and an episode might undergo more significant structural remodeling in these areas after exposure to SLEs, potentially as a maladaptive response to stress [10]. Previous meta-analyses on cross-sectional studies have associated CM with reduced gray matter in the same regions [11, 12]. Interestingly, this interaction was not observed in MDD patients without an episode, despite similar levels of experienced SLEs (see Supplementary Table S9), nor in HCs, indicating potential neurobiological adaptive mechanisms in response to CM and recent stress in these groups.

While the prevailing literature associates stress and depression with GMV reductions in the dorsolateral prefrontal cortex (DLPFC), anterior cingulate cortex, and hippocampus [49], potentially due to a loss in synaptic density [50–52], our study found a longitudinal GMV increase in MDD patients with an episode within the middle frontal gyrus– a DLPFC region known for its structural plasticity and connection to the limbic system [36, 53]. This finding aligns with the observed three-way interaction with hsCRP (see Supplementary Results 3), suggesting that this GMV increase may be attributable to mechanisms like glial cell proliferation, particularly of astrocytes and microglia [54–56], potentially due to low-grade neuroinflammation and increased blood-brain barrier permeability [56–60]. The observed GMV increases could thus reflect maladaptive biological responses to recent stress, such as the recruitment of additional capillaries and glial cells to meet the increased metabolic demands [59, 61]. Over time, these adaptations may lead to sustained GMV reductions, as often found in case-control MRI studies in MDD, potentially through synaptic pruning and consecutive neural shrinkage induced by glial cell proliferation [62, 63]. Our findings underscore the complex interplay of pre-existing vulnerabilities (CM or elevated hsCRP), external stressors (SLEs), and the recurrence of MDD episodes on brain structure – a relationship this study is the first to investigate.

Some limitations of this study must be noted. First, the measures of SLEs and CM were self-reported, which could have introduced recall bias and measurement error [64]; yet, instruments such as the Life Events Questionnaire (LEQ) or the Childhood Trauma Questionnaire (CTQ) are known to be state-independent in MDD patients and stable over time [65, 66]. Second, although we accounted for many potential confounders, such as clinical and psychosocial variables, other factors, like physical health or lifestyle factors [67] could have influenced the observed relationships, precluding causal inferences.

Our findings suggest distinct patterns of GMV change in response to SLEs between MDD patients and HCs across the middle frontal, precentral, and postcentral gyri. GMV alterations in HCs might represent adaptive responses to stress, while GMV alterations in MDD patients with a history of CM and recent depressive episodes could indicate maladaptive changes. Interestingly, this model seems to uniquely impact brain structure in MDD patients who experienced an episode during our 2-year investigational interval, suggesting a potential neural foundation for the diathesis-stress model in MDD recurrences. Our study underscores the importance of using a comprehensive and longitudinal approach to gain a better understanding of the pathomechanisms behind MDD. Future studies should elucidate the observed relationships with even longer follow-up periods, multiple assessment points, and replication in other psychiatric disorders, such as schizophrenia or bipolar disorder.

## Supplementary information

Supplementary Online Content

## Data Availability

The data that support the findings of this study are available from the corresponding author (FTO) upon reasonable request. MATLAB (version R2017a) code was used to generate batches for whole-brain analyses within the SPM12/CAT12 toolbox and are available from the corresponding author (FTO) upon reasonable request.

## References

[1] Cohen S, Murphy MLM, Prather AA. Ten surprising facts about stressful life events and disease risk. Annu Rev Psychol. 2019;70:577–97. 29949726 10.1146/annurev-psych-010418-102857 PMC6996482

[2] Monroe SM, Simons AD. Diathesis-stress theories in the context of life stress research: Implications for the depressive disorders. Psychol Bull. 1991;110:406–25. 1758917 10.1037/0033-2909.110.3.406

[3] Wilde A, Chan HN, Rahman B, Meiser B, Mitchell PB, Schofield PR, et al. A meta-analysis of the risk of major affective disorder in relatives of individuals affected by major depressive disorder or bipolar disorder. J Affect Disord. 2014;158:37–47. 24655763 10.1016/j.jad.2014.01.014

[4] Nelson J, Klumparendt A, Doebler P, Ehring T. Childhood maltreatment and characteristics of adult depression: meta-analysis. Br J Psychiatry. 2017;210:96–104. 27908895 10.1192/bjp.bp.115.180752

[5] Buckman JEJ, Underwood A, Clarke K, Saunders R, Hollon SD, Fearon P, et al. Risk factors for relapse and recurrence of depression in adults and how they operate: a four-phase systematic review and meta-synthesis. Clin Psychol Rev. 2018;64:13–38. 30075313 10.1016/j.cpr.2018.07.005 PMC6237833

[6] Stroud CB, Davila J, Hammen C, Vrshek-Schallhorn S. Severe and nonsevere events in first onsets versus recurrences of depression: evidence for stress sensitization. J Abnorm Psychol. 2011;120:142–54. 21171724 10.1037/a0021659

[7] Ansell EB, Rando K, Tuit K, Guarnaccia J, Sinha R. Cumulative adversity and smaller gray matter volume in medial prefrontal, anterior cingulate, and insula regions. Biol Psychiatry. 2012;72:57–64. 22218286 10.1016/j.biopsych.2011.11.022 PMC3391585

[8] Kuhn M, Scharfenort R, Schümann D, Schiele MA, Münsterkötter AL, Deckert J, et al. Mismatch or allostatic load? Timing of life adversity differentially shapes gray matter volume and anxious temperament. Soc Cogn Affect Neurosci. 2016;11:537–47. 26568620 10.1093/scan/nsv137 PMC4814783

[9] Ringwald KG, Meller T, Schmitt S, Andlauer TFM, Stein F, Brosch K, et al. Interaction of developmental factors and ordinary stressful life events on brain structure in adults. Neuroimage Clin. 2021;30:102683. 34215153 10.1016/j.nicl.2021.102683 PMC8102615

[10] Ringwald KG, Pfarr JK, Schmitt S, Stein F, Brosch K, Meller T, et al. Interaction of recent stressful life events and childhood abuse on orbitofrontal grey matter volume in adults with depression. J Affect Disord. 2022;312:122–7. 35753498 10.1016/j.jad.2022.06.050

[11] Yang W, Jin S, Duan W, Yu H, Ping L, Shen Z, et al. The effects of childhood maltreatment on cortical thickness and gray matter volume: a coordinate-based meta-analysis. Psychol Med. 2023;53:1681–99. 36946124 10.1017/S0033291723000661

[12] Paquola C, Bennett MR, Lagopoulos J. Understanding heterogeneity in grey matter research of adults with childhood maltreatment—a meta-analysis and review. Neurosci Biobehav Rev. 2016;69:299–312. 27531235 10.1016/j.neubiorev.2016.08.011

[13] Teicher MH, Samson JA, Anderson CM, Ohashi K. The effects of childhood maltreatment on brain structure, function and connectivity. Nat Rev Neurosci. 2016;17:652–66. 27640984 10.1038/nrn.2016.111

[14] Ringwald KG, Pfarr JK, Stein F, Brosch K, Meller T, Thomas-Odenthal F, et al. Association between stressful life events and grey matter volume in the medial prefrontal cortex: a 2-year longitudinal study. Hum Brain Mapp. 2022;43:3577–84. 35411559 10.1002/hbm.25869 PMC9248310

[15] Papagni SA, Benetti S, Arulanantham S, McCrory E, McGuire P, Mechelli A. Effects of stressful life events on human brain structure: A longitudinal voxel-based morphometry study. Stress. 2011;14:227–32. 21034297 10.3109/10253890.2010.522279

[16] Kircher T, Wöhr M, Nenadic I, Schwarting R, Schratt G, Alferink J, et al. Neurobiology of the major psychoses: a translational perspective on brain structure and function—the FOR2107 consortium. Eur Arch Psychiatry Clin Neurosci. 2019;269:949–62. 30267149 10.1007/s00406-018-0943-x

[17] Wittchen H-U, Wunderlich U, Gruschwitz S, Zaudig M. SKID I. Strukturiertes Klinisches Interview für DSM-IV. Achse I: Psychische Störungen. Interviewheft und Beurteilungsheft. Eine deutschsprachige, erweiterte Bearb. d. amerikanischen Originalversion des SKID I. Göttingen: Hogrefe; 1997.

[18] Spielberger CD, Gorsuch RL, Lushene RE. Manual for the state-trait anxiety inventory (self-evaluation questionnaire). Consulting Psychol: Palo Alto, 1970.

[19] Hamilton M. A rating scale for depression. J Neurol Neurosurg Psychiatry. 1960;23:56. 14399272 10.1136/jnnp.23.1.56 PMC495331

[20] Hall RCW. Global assessment of functioning: a modified scale. Psychosomatics. 1995;36:267–75. 7638314 10.1016/S0033-3182(95)71666-8

[21] Cohen S, Kamarck T, Mermelstein R. A global measure of perceived stress. J Health Soc Behav. 1983;24:385–96. 6668417

[22] Costa PT, McCrae RR. Normal personality assessment in clinical practice: the NEO personality inventory. Psychol Assess. 1992;4:5.

[23] Leppert K, Koch B, Brähler E, Strauß B. Die Resilienzskala (RS)-Überprüfung der Langform RS-25 und einer Kurzform RS-13. 2008.

[24] Fydrich T, Geyer M, Hessel A, Sommer G, Brähler E. Fragebogen zur Sozialen Unterstützung (F-SozU): Normierung an einer repräsentativen Stichprobe. Diagnostica. 1999;45:212–6.

[25] Steffanowski A, Oppl M, Meyerberg J, Schmidt J, Wittmann WW, Nübling R. Psychometrische Überprüfung einer deutschsprachigen version des relationship scales questionnaire (RSQ). In: Störungsspezifische Therapieansätze-Konzepte Und Ergebnisse. Gießen: Psychosozial Verlag; 2001, pp. 320–342.

[26] Wingenfeld K, Spitzer C, Mensebach C, Grabe HJ, Hill A, Gast U, et al. The german version of the Childhood Trauma Questionnaire (CTQ): preliminary psychometric properties. Psychother Psychosom Med Psychol. 2010;60:442–50. 20200804 10.1055/s-0030-1247564

[27] Norbeck JS. Modification of life event questionnaires for use with female respondents. Res Nurs Health. 1984;7:61–71. 6565302 10.1002/nur.4770070110

[28] Vogelbacher C, Möbius TWD, Sommer J, Schuster V, Dannlowski U, Kircher T, et al. The Marburg-Münster affective disorders cohort study (MACS): a quality assurance protocol for MR neuroimaging data. Neuroimage. 2018;172:450–60. 29410079 10.1016/j.neuroimage.2018.01.079

[29] Kuhn L, Noack H, Wagels L, Prothmann A, Schulik A, Aydin E, et al. Sex-dependent multimodal response profiles to psychosocial stress. Cerebral Cortex. 2023;33:583–96. 35238348 10.1093/cercor/bhac086

[30] Taki Y, Kinomura S, Sato K, Goto R, Kawashima R, Fukuda H. A longitudinal study of gray matter volume decline with age and modifying factors. Neurobiol Aging. 2011;32:907–15. 19497638 10.1016/j.neurobiolaging.2009.05.003

[31] Jamovi Project. Jamovi (Version 2.3. 18) [Computer Software]. 2021.

[32] Steiger JH. Tests for comparing elements of a correlation matrix. Psychol Bull. 1980;87:245.

[33] Briggs RG, Lin Y-H, Dadario NB, Kim SJ, Young IM, Bai MY, et al. Anatomy and white matter connections of the middle frontal Gyrus. World Neurosurg. 2021;150:e520–9. 33744423 10.1016/j.wneu.2021.03.045

[34] Caetano I, Ferreira S, Coelho A, Amorim L, Castanho TC, Portugal-Nunes C, et al. Perceived stress modulates the activity between the amygdala and the cortex. Mol Psychiatry. 2022;27:4939–47. 36117211 10.1038/s41380-022-01780-8

[35] Goldfarb EV, Rosenberg MD, Seo D, Constable RT, Sinha R. Hippocampal seed connectome-based modeling predicts the feeling of stress. Nat Commun. 2020;11:2650. 32461583 10.1038/s41467-020-16492-2 PMC7253445

[36] Hossein S, Cooper JA, DeVries BAM, Nuutinen MR, Hahn EC, Kragel PA, et al. Effects of acute stress and depression on functional connectivity between prefrontal cortex and the amygdala. Mol Psychiatry. 19 April 2023. 10.1038/s41380-023-02056-5. 10.1038/s41380-023-02056-5 PMC11887625 37076616

[37] Phelps EA. Human emotion and memory: interactions of the amygdala and hippocampal complex. Curr Opin Neurobiol. 2004;14:198–202. 15082325 10.1016/j.conb.2004.03.015

[38] Kropf E, Syan SK, Minuzzi L, Frey BN. From anatomy to function: the role of the somatosensory cortex in emotional regulation. Braz J Psychiatry. 2019;41:261–9. 30540029 10.1590/1516-4446-2018-0183 PMC6794131

[39] Banker L, Tadi P. Neuroanatomy, Precentral Gyrus. Treasure Island (FL): StatPearls Publishing; 2023. 31334938

[40] Hermans EJ, Henckens MJAG, Joëls M, Fernández G. Dynamic adaptation of large-scale brain networks in response to acute stressors. Trends Neurosci. 2014;37:304–14. 24766931 10.1016/j.tins.2014.03.006

[41] van Leeuwen JMC, Vink M, Fernández G, Hermans EJ, Joëls M, Kahn RS, et al. At-risk individuals display altered brain activity following stress. Neuropsychopharmacology. 2018;43:1954–60. 29483659 10.1038/s41386-018-0026-8 PMC6046038

[42] Fischer AS, Ellwood-Lowe ME, Colich NL, Cichocki A, Ho TC, Gotlib IH. Reward-circuit biomarkers of risk and resilience in adolescent depression. J Affect Disord. 2019;246:902–9. 30795497 10.1016/j.jad.2018.12.104 PMC6391738

[43] Fischer AS, Hagan KE, Gotlib IH. Functional neuroimaging biomarkers of resilience in major depressive disorder. Curr Opin Psychiatry. 2021;34:22–28. 33027183 10.1097/YCO.0000000000000662 PMC7769009

[44] Park HRP, Quidé Y, Schofield PR, Williams LM, Gatt JM. Grey matter covariation and the role of emotion reappraisal in mental wellbeing and resilience after early life stress exposure. Transl Psychiatry. 2022;12:85. 35220403 10.1038/s41398-022-01849-6 PMC8882193

[45] Shi Y, Bai Y, Zhang L, Chen Y, Liu X, Liu Y, et al. Psychological resilience mediates the association of the middle frontal gyrus functional connectivity with sleep quality. Brain Imaging Behav. 2022;16:2735–43. 36307619 10.1007/s11682-022-00735-5

[46] Li X, Zhang M, Li K, Zou F, Wang Y, Wu X, et al. The altered somatic brain network in state anxiety. Front Psychiatry. 2019;10:465. 31312147 10.3389/fpsyt.2019.00465 PMC6613038

[47] Michalski LJ, Demers CH, Baranger DAA, Barch DM, Harms MP, Burgess GC, et al. Perceived stress is associated with increased rostral middle frontal gyrus cortical thickness: a family‐based and discordant‐sibling investigation. Genes Brain Behav. 2017;16:781–9. 28749606 10.1111/gbb.12404 PMC5677535

[48] Hammen C. Stress and depression. Annu Rev Clin Psychol. 2005;1:293–319. 17716090 10.1146/annurev.clinpsy.1.102803.143938

[49] Marx W, Penninx BWJH, Solmi M, Furukawa TA, Firth J, Carvalho AF, et al. Major depressive disorder. Nat Rev Dis Primers. 2023;9:44. 37620370 10.1038/s41572-023-00454-1

[50] Duman RS, Aghajanian GK, Sanacora G, Krystal JH. Synaptic plasticity and depression: new insights from stress and rapid-acting antidepressants. Nat Med. 2016;22:238–49. 26937618 10.1038/nm.4050 PMC5405628

[51] Holmes SE, Scheinost D, Finnema SJ, Naganawa M, Davis MT, DellaGioia N, et al. Lower synaptic density is associated with depression severity and network alterations. Nat Commun. 2019;10:1529. 30948709 10.1038/s41467-019-09562-7 PMC6449365

[52] Kassem MS, Lagopoulos J, Stait-Gardner T, Price WS, Chohan TW, Arnold JC, et al. Stress-induced grey matter loss determined by MRI is primarily due to loss of dendrites and their synapses. Mol Neurobiol. 2013;47:645–61. 23138690 10.1007/s12035-012-8365-7

[53] Opel N, Cearns M, Clark S, Toben C, Grotegerd D, Heindel W, et al. Large-scale evidence for an association between low-grade peripheral inflammation and brain structural alterations in major depression in the BiDirect study. J Psychiatry Neurosci. 2019;44:423–31. 31304733 10.1503/jpn.180208 PMC6821515

[54] Femminella GD, Dani M, Wood M, Fan Z, Calsolaro V, Atkinson R, et al. Microglial activation in early Alzheimer trajectory is associated with higher gray matter volume. Neurology. 2019;92:e1331–43. 30796139 10.1212/WNL.0000000000007133 PMC6511099

[55] Asan L, Falfán-Melgoza C, Beretta CA, Sack M, Zheng L, Weber-Fahr W, et al. Cellular correlates of gray matter volume changes in magnetic resonance morphometry identified by two-photon microscopy. Sci Rep. 2021;11:4234. 33608622 10.1038/s41598-021-83491-8 PMC7895945

[56] Green C, Shen X, Stevenson AJ, Conole ELS, Harris MA, Barbu MC, et al. Structural brain correlates of serum and epigenetic markers of inflammation in major depressive disorder. Brain Behav Immun. 2021;92:39–48. 33221487 10.1016/j.bbi.2020.11.024 PMC7910280

[57] Gritti D, Delvecchio G, Ferro A, Bressi C, Brambilla P. Neuroinflammation in major depressive disorder: a review of PET imaging studies examining the 18-kDa translocator protein. J Affect Disord. 2021;292:642–51. 34153835 10.1016/j.jad.2021.06.001

[58] Goldsmith DR, Bekhbat M, Mehta ND, Felger JC. Inflammation-related functional and structural dysconnectivity as a pathway to psychopathology. Biol Psychiatry. 2023;93:405–18. 36725140 10.1016/j.biopsych.2022.11.003 PMC9895884

[59] Setiawan E, Wilson AA, Mizrahi R, Rusjan PM, Miler L, Rajkowska G, et al. Role of translocator protein density, a marker of neuroinflammation, in the brain during major depressive episodes. JAMA Psychiatry. 2015;72:268. 25629589 10.1001/jamapsychiatry.2014.2427 PMC4836849

[60] Welcome MO. Cellular mechanisms and molecular signaling pathways in stress-induced anxiety, depression, and blood–brain barrier inflammation and leakage. Inflammopharmacology. 2020;28:643–65. 32333258 10.1007/s10787-020-00712-8

[61] Uhlig M, Reinelt JD, Lauckner ME, Kumral D, Schaare HL, Mildner T, et al. Rapid volumetric brain changes after acute psychosocial stress. Neuroimage. 2023;265:119760. 36427754 10.1016/j.neuroimage.2022.119760

[62] Deng S, Chen J, Wang F. Microglia: a central player in depression. Curr Med Sci. 2020;40:391–400. 32681244 10.1007/s11596-020-2193-1

[63] Ren F, Guo R. Synaptic microenvironment in depressive disorder: insights from synaptic plasticity. Neuropsychiatr Dis Treat. 2021;17:157–65. 33519203 10.2147/NDT.S268012 PMC7838013

[64] Coughlin SS. Recall bias in epidemiologic studies. J Clin Epidemiol. 1990;43:87–91. 2319285 10.1016/0895-4356(90)90060-3

[65] Brugha TS, Cragg D. The List of Threatening Experiences: the reliability and validity of a brief life events questionnaire. Acta Psychiatr Scand. 1990;82:77–81. 2399824 10.1111/j.1600-0447.1990.tb01360.x

[66] Goltermann J, Meinert S, Hülsmann C, Dohm K, Grotegerd D, Redlich R, et al. Temporal stability and state-dependence of retrospective self-reports of childhood maltreatment in healthy and depressed adults. Psychol Assess. 2023;35:12–22. 10.1037/pas0001175 36355690

[67] McEwen BS. Protective and damaging effects of stress mediators: central role of the brain. Dialogues Clin Neurosci. 2006;8:367–81. 17290796 10.31887/DCNS.2006.8.4/bmcewen PMC3181832

