# Supplementary material for: Neural foundation of the diathesis-stress model: longitudinal gray matter volume changes in response to stressful life events in major depressive disorder and healthy controls
Source: Mol Psychiatry. 2024 Mar 29;29(9):2724–32. doi: 10.1038/s41380-024-02526-4 (PMC11420061; doi:10.1038/s41380-024-02526-4)
Supplement: Supplementary file 1 — Supplementary Online Content [file 41380_2024_2526_MOESM1_ESM.docx]

**Supplementary Online Content**

**Supplement 1.** Rationale for inclusion of C-reactive protein (CRP) in exploratory analyses

**Supplementary Methods.** Assessment of high-sensitivity CRP (hsCRP)

**Table S1:** Results from whole-brain analyses FWE corrected for cluster-level significance

**Table S2:** Effect of current medication intake (yes/no) at follow-up (T2) time point on brain structural changes related to stressful life events (LEQ) during the two-year interval (T2-T1) within MDD patients

**Table S3**: Relationship between clinical-psychological variables at follow-up (T2) time point and GMV changes related to stressful life events (LEQ) during the two-year interval (T2-T1) among groups

**Supplementary Results 1.** Differences in correlation coefficients of positive and negative stressful life events (LEQ) on GMV change (T2-T1) using Steiger’s Z test

**Table S4:** Model coefficients of three-way interactions between stressful life events (LEQ), childhood maltreatment (CTQ), and group (MDD vs. HC) on GMV changes (T2-T1) in the precentral / postcentral and middle frontal gyri (LEQ x CTQ x group)

**Table S5**: Three-way interactions between stressful life events (LEQ) and clinical-psychosocial factors at follow-up (T2) time point on GMV changes (T2-T1) among groups (LEQ x factor x group)

**Supplementary Results 2.** Results from exploratory whole-brain analyses FWE corrected for cluster-level significant on the association between SLEs and GMV change in MDD recurrence and HC

**Figure S1.** Association between stressful life events (LEQ) and GMV change between MDD patients with and without an episode and HCs during the two-year interval (T2-T1)

**Table S6:** Model coefficients of three-way interactions between stressful life events (LEQ), childhood maltreatment (CTQ), and at least one depressive episode (yes vs. no vs. HC) on GMV changes (T2-T1) in the left middle frontal / precentral / postcentral gyri (LEQ x CTQ x recurrence group)

**Supplementary Results 3**. Moderation of hsCRP on the association between SLEs and GMV change

**Figure S2.** Three-way interaction between stressful life events (LEQ) and baseline (T1) high-sensitivity C-reactive protein (hsCRP) on GMV change in the middle frontal / precentral / postcentral gyri between MDD patients with and without an episode during the two-year interval and HCs

**Table S7:** Model coefficients of three-way interactions between stressful life events (LEQ), baseline (T1) high-sensitivity C-reactive protein (hsCRP), and at least one depressive episode (yes vs. no vs. HC) on GMV changes (T2-T1) in the left middle frontal / precentral / postcentral gyri (LEQ x hsCRP x recurrence group)

**Table S8:** Model coefficients of three-way interactions between stressful life events (LEQ), baseline (T1) high-sensitivity C-reactive protein (hsCRP), and group (MDD vs. HC) on GMV changes (T2-T1) in the middle frontal / precentral / postcentral gyri (LEQ x hsCRP x group)

**Table S9:** Descriptive statistics of HC, MDD non-recurrence, and MDD recurrence group at baseline (T1) and follow-up (T2) time points

**Table S10**: Three-way interactions between stressful life events (LEQ), childhood maltreatment, and clinical factors during the two-year interval on GMV change (T2-T1) in MDD patients (LEQ x CTQ x factor)

**Table S11**: Three-way interactions between stressful life events (LEQ) and clinical-psychosocial factors at follow-up (T2) on GMV changes (T2-T1) in MDD patients with and without an episode and HCs (LEQ x factors x recurrence group)

**Figure S3.** Predictive association between cumulative stressful life events (LEQ total events score) during the two-year follow-up period (T2-T1) and baseline (T1) GMV in the precentral / postcentral gyri among groups

**Figure S4.** Cluster overlap of predictive baseline (T1; red) and longitudinal (T2-T1; blue) associations between stressful life events (LEQ) and GMV in the precentral / postcentral gyri

**References**

**Supplement 1. Rationale for inclusion of C-reactive protein (CRP) in exploratory analyses**

Low-grade inflammatory processes may modulate such GMV changes [1–3]. Stress and the onset and recurrence of depression have been linked to elevated peripheral inflammatory markers, such as C-reactive protein (CRP) [4–6]. Inflammatory processes can affect the brains’ activity of glial cells, such as microglia and astrocytes [7–10], whose proliferation or dysfunction can lead to GMV changes [11, 12]. Such changes may occur through synaptic refinement or pruning [13], particularly in regions like the hippocampus and prefrontal cortex, which are critical for stress responses and emotion regulation [14]. Elucidating the relationship between CRP, SLEs, and GMV may shed light onto the underlying biological mechanisms of the GMV changes.

**Supplementary Methods.**

**Assessment of high-sensitivity CRP (hsCRP).** HsCRP data were available from 509 participants (67.5%). Blood samples were taken at baseline (T1) from non-fasting participants using tubes with additives to collect heparin plasma. At both the Marburg and Münster sites, samples were frozen at -80° Celsius within 1.5 hours of collection. The Marburg samples were directly stored at the Comprehensive Biomaterial Bank Marburg (CBBMR), and the Münster samples were transported to the CBBMR for centralized processing. All samples were centrifuged and analyzed on an AU5800 clinical chemistry analyzer (Beckman Coulter) using a CRP Latex Highly Sensitive Calibrator (Cat#ODC0027), which determines CRP turbidimetrically. This high-sensitive test has a detection limit of 0.08 mg/l, with the normal concentration for this assay being less than 1 mg/L. Human CRP reacts specifically with antibodies bound to latex particles, generating insoluble aggregates. The absorption of these aggregates is proportional to the CRP concentration of the sample. The routine inter-assay coefficient of variation was consistently below 5%. Fourteen participants (2.75%) had values below this limit but were included in the analysis. Across the whole sample, hsCRP values ranged from 0.08 to 44.77 mg/L (mean = 2.27, SD = 4.40). Twelve participants were excluded from the analysis due to hsCRP values greater than 3 SD above the mean (>15.47 mg/L), which might indicate acute inflammatory processes.

**Statistical analysis.** For hsCRP analyses, covariates such as body mass index (BMI), nonsteroidal anti-inflammatory drug (NSAID) use, charge/box number, and current smoking status (yes/no) were added in addition to the covariates of the main analyses.

| **Table S1:** Results from whole-brain analyses FWE corrected for cluster-level significance | | | | | | | | | |
| --- | --- | --- | --- | --- | --- | --- | --- | --- | --- |
|  |  | MNI coordinates | | |  |  |  |  |  |
|  | **H** | **x** | **y** | **z** | ***T*** | ***k***  **cluster** | **Cohen’s**  ***d*** | ***p***  **FWE cluster-level** |  |
|  | **HC < MDD (slope)** | | | | | | | | |
|  |  |  |  |  |  |  |  |  |  |
| 54% Postcentral gyrus  31% Precentral gyrus  15% Supramarginal gyrus | L | -52 | -22 | 44 | 4.50 | 1364 | 0.33 | .009 |  |
|  |  |  |  |  |  |  |  |  |  |
| 98% Middle frontal gyrus | L | -46 | 27 | 28 | 4.02 | 1588 | 0.29 | .005 |  |
|  |  | | | | | | | | |
| *Note*: R=right, L=left, H=hemisphere; *k*, number of significant voxels per cluster after adjustment for multiple testing (i.e., FWE cluster-level correction). Percentages show to what extent the identified clusters lie in the brain regions of the Dartel space Neuromorphometrics atlas. | | | | | | | | | |

| **Table S2:** Effect of current medication intake (yes/no) at follow-up (T2) time point on brain structural changes related to stressful life events (LEQ) during the two-year interval (T2-T1) within MDD patients | | | |
| --- | --- | --- | --- |
|  | | **Left precentral/**  **postcentral gyri** | **Left middle**  **frontal gyrus** |
| **Antidepressants** | *F* | 1.76 | 0.18 |
|  | *p* | .185 | .669 |
|  | *df* | 354 | 354 |
| **Antipsychotics** | *F* | 3.99 | 3.40 |
|  | *p* | .047 | .066 |
|  | *df* | 354 | 354 |
| **Lithium** | *F* | 0.34 | 0.07 |
|  | *p* | .561 | .785 |
|  | *df* | 354 | 354 |
| *Note.* Using ANCOVA, results indicate that medication intake had no influence on the identified clusters, after correction for multiple testing. Levene's test indicated homogeneity of variance across groups for all analyses (*p*>.05). | | | |

| **Table S3**: Relationship between clinical-psychological variables at follow-up (T2) time point and GMV changes related to stressful life events (LEQ) during the two-year interval (T2-T1) among groups | | | | | |
| --- | --- | --- | --- | --- | --- |
|  | | **Left precentral/ postcentral gyri** | | **Left middle**  **frontal gyrus** | |
|  |  | **HC** | **MDD** | **HC** | **MDD** |
| **Number of depressive episodes during interval** | *rho* | – | -0.01 | – | 0.01 |
|  | *p* | – | .994 | – | .796 |
|  | *N* | – | 357 | – | 357 |
| **Duration of hospitalization during interval** | *rho* | – | -0.07 | – | -0.11 |
|  | *p* | – | .185 | – | .035 |
|  | *N* | – | 353 | – | 353 |
| **Remission status** | *F* | – | 0.04 | – | 0.44 |
|  | *p* | – | .844 | – | .507 |
|  | *df* | – | 354 | – | 354 |
| **Family history of MDD/BD/SCZ/SZA** | *F* | 0.14 | 0.00 | 0.47 | 1.23 |
|  | *p* | .712 | .952 | .491 | .268 |
|  | *df* | 382 | 347 | 382 | 347 |
| **PSS** | *r* | 0.02 | 0.04 | 0.02 | 0.04 |
|  | *p* | .676 | .487 | .755 | .412 |
|  | *N* | 381 | 348 | 381 | 348 |
| **NEO-FFI neuroticism** | *r* | 0.04 | 0.03 | 0.10 | 0.08 |
|  | *p* | .383 | .592 | .059 | .134 |
|  | *N* | 386 | 357 | 386 | 357 |
|  | *r* | -0.03 | 0.01 | -0.04 | 0.00 |
| **GAF** | *p* | .601 | .830 | .413 | .960 |
|  | *N* | 384 | 360 | 384 | 360 |
| **STAI-S** | *r* | -0.02 | 0.03 | -0.07 | 0.07 |
|  | *p* | .720 | .599 | .177 | .190 |
|  | *N* | 383 | 349 | 383 | 349 |
| **HAM-D** | *rho* | 0.12 | 0.02 | 0.03 | 0.01 |
|  | *p* | .015 | .776 | .509 | .730 |
|  | *N* | 387 | 357 | 387 | 357 |
|  | *rho* | -0.01 | -0.03 | 0.05 | -0.08 |
| **CTQ** | *p* | .911 | .604 | .327 | .134 |
|  | *N* | 385 | 350 | 385 | 350 |
| *Note*. CTQ, childhood trauma questionnaire; GAF, Global Assessment of Functioning; HAM-D, Hamilton Depression Rating Scale; NEOFFI, NEO Five-Factor Inventory questionnaire; PSS, Perceived Stress Scale questionnaire; STAI-S, State-Trait Anxiety Inventory. Using partial correlations (Pearson or Spearman’s rho if non-normal) and ANCOVA, we assessed the relationship between cluster intensity values and potential confounders in the connection between stressful life events and GMV change. Results indicate that the alterations were not influenced by factors other than stressful life events on the brain. | | | | | |

**Supplementary Results 1.** **Differences in correlation coefficients of positive and negative stressful life events (LEQ) on GMV change (T2-T1) using Steiger’s Z test**

**Healthy controls.** Overall, there was a negative correlation between positive stressful life events (SLEs) and GMV change in the precentral/postcentral gyri (*rho*(392)= -0.12, *p*=.018), and middle frontal gyrus (*rho*(392)= -0.10, *p*=.047). There was also a negative correlation between negative SLEs and GMV change in the precentral/postcentral gyri (*rho*(392)= -0.20, *p*<.001) and middle frontal gyrus (*rho*(392)= -0.19, *p*<.001). However, correlations of positive and negative SLEs were not significantly different with respect to GMV change (precentral/postcentral gyri: *t*(392)=1.36, *p*=.174; middle frontal gyrus: *t*(392)=1.53, *p*=.126). Thus, the impact on GMV changes in these areas was consistent between positive and negative SLEs in HCs, regardless of its valence.

**MDD patients.** Overall, there was no significant correlation between positive SLEs and GMV change in the precentral/postcentral gyri (*rho*(362)= -0.01, *p*=.906), and middle frontal gyrus (*rho*(362)= 0.06, *p*=.272). There was also no significant correlation between negative SLEs and GMV change in the precentral/postcentral gyri (*rho*(362)= 0.10, *p*<.071) and middle frontal gyrus (*rho*(362)= 0.08, *p*<.131). Correlations of positive and negative SLEs were not significantly different with respect to GMV change (precentral/postcentral gyri: *t*(362)=-1.55, *p*=.122; middle frontal gyrus: *t*(362)=-0.41, *p*=.682).

| **Table S4:** Model coefficients of three-way interactions between stressful life events (LEQ), childhood maltreatment (CTQ), and group (MDD vs. HC) on GMV changes (T2-T1) in the precentral / postcentral and middle frontal gyri (LEQ x CTQ x group) | | | | | |
| --- | --- | --- | --- | --- | --- |
| **Predictor** | ***β*** | ***B*** | **SE** | ***t*** | ***p*** |
| **Left precentral/postcentral gyri** |  |  |  |  |  |
| Intercept |  | -0.01 | 0.01 | -0.75 | .456 |
| Interscan interval | 0.01 | 0.00 | 0.00 | 0.33 | .741 |
| Body-coil change | -0.10 | -0.00 | 0.00 | -2.67 | .008 |
| Gradient-coil change | -0.05 | -0.00 | 0.00 | -1.32 | .188 |
| Age | 0.05 | 0.00 | 0.00 | 1.31 | .189 |
| Sex | 0.10 | 0.00 | 0.00 | 2.74 | .006 |
| LEQ | -0.09 | -0.00 | 0.00 | -0.60 | .552 |
| CTQ | 0.01 | 0.00 | 0.00 | 1.04 | .298 |
| Group | 0.03 | 0.00 | 0.01 | 0.09 | .927 |
| LEQ ✻ CTQ | -0.03 | -0.00 | 0.00 | -0.55 | .585 |
| LEQ ✻ Group | 0.20 | 0.00 | 0.00 | 0.72 | .472 |
| CTQ ✻ Group | -0.06 | -0.00 | 0.00 | -1.08 | .280 |
| LEQ ✻ CTQ ✻ Group | 0.03 | 0.00 | 0.00 | 0.52 | .604 |
| **Left middle frontal gyrus** |  |  |  |  |  |
| Intercept |  | -0.01 | 0.01 | -0.82 | .411 |
| Interscan interval | -0.02 | -0.00 | 0.00 | -0.51 | .609 |
| Body-coil change | -0.02 | -0.00 | 0.00 | -0.54 | .589 |
| Gradient-coil change | -0.09 | -0.00 | 0.00 | -2.26 | .024 |
| Age | 0.08 | 0.00 | 0.00 | 2.02 | .044 |
| Sex | 0.07 | 0.00 | 0.00 | 2.02 | .043 |
| LEQ | -0.05 | -0.00 | 0.00 | -1.51 | .132 |
| CTQ | 0.03 | 0.00 | 0.00 | 0.78 | .434 |
| Group | 0.09 | 0.00 | 0.01 | 0.62 | .534 |
| LEQ ✻ CTQ | 0.05 | 0.00 | 0.00 | 0.55 | .585 |
| LEQ ✻ Group | 0.16 | 0.00 | 0.00 | 1.43 | .154 |
| CTQ ✻ Group | -0.15 | -0.00 | 0.00 | -1.13 | .260 |
| LEQ ✻ CTQ ✻ Group | -0.02 | -0.00 | 0.00 | -0.41 | .681 |
| *Note.* Precentral/postcentral gyri: *F*(12,722)= 3.78, *p*<.001; middle frontal gyrus: *F*(12,722)=4.42, *p*<.001. | | | | | |

| **Table S5**: Three-way interactions between stressful life events (LEQ) and clinical-psychosocial factors at follow-up (T2) time point on GMV changes (T2-T1) among groups (LEQ x factor x group) | | | |
| --- | --- | --- | --- |
|  | | **Left precentral/ postcentral gyri** | **Left middle**  **frontal gyrus** |
| **Number of depressive episodes during interval ^+^** | *β* | 0.05 | 0.08 |
|  | *p* | .376 | .203 |
|  | *t* | 0.89 | 1.28 |
| **Duration of hospitalization during interval ^+^** | *β* | -0.00 | 0.02 |
|  | *p* | .982 | .700 |
|  | *t* | -0.02 | 0.39 |
| **Family history of MDD/BD/SCZ/SZA** | *β* | 0.04 | 0.04 |
|  | *p* | .280 | .320 |
|  | *t* | 1.08 | 0.99 |
| **HAM-D** | *β* | 0.03 | 0.07 |
|  | *p* | .693 | .443 |
|  | *t* | 0.39 | 0.77 |
| **STAI-S** | *β* | 0.08 | 0.05 |
|  | *p* | .113 | .299 |
|  | *t* | 1.59 | 1.04 |
| **NEO-FFI neuroticism** | *β* | -0.02 | -0.04 |
|  | *p* | .771 | .474 |
|  | *t* | -0.29 | -0.72 |
| **RS25** | *β* | -0.11 | -0.05 |
|  | *p* | .036 | .327 |
|  | *t* | -2.10 | -0.98 |
| **FSozU/SSQ** | *β* | -0.06 | 0.00 |
|  | *p* | .292 | .985 |
|  | *t* | -1.05 | 0.02 |
| **Secure childhood attachment (RSQ)** | *β* | 0.03 | 0.03 |
|  | *p* | .502 | .504 |
|  | *t* | 0.67 | 0.67 |
| *Note*. BD, bipolar disorder; FSozU/SSQ, social support questionnaire; HAM-D, Hamilton Depression Rating Scale; LEQ, Life Events Questionnaire; MDD, major depressive disorder; NEOFFI, NEO Five-Factor Inventory questionnaire; RS25, 25-item Resilience Scale; RSQ, Relationship Scales Questionnaire; SZA, schizoaffective disorder; SCZ, schizophrenia; STAI-S, State-Trait Anxiety Inventory. Using multiple linear regression, we assessed the interactions between cluster intensity values and confounders in the relationship between stressful life events and GMV change. Results indicate that no other variables, next to childhood maltreatment, significantly interacted with stressful life events to influence GMV changes, after correction for multiple testing. ^+^These variables were tested in two-way interactions in MDD patients. | | | |

**Supplementary Results 2. Results from exploratory whole-brain analyses FWE corrected for cluster-level significance on the association between SLEs and GMV change in MDD recurrence, MDD non-recurrence, and HC**

**Exploratory whole-brain analyses**. To disentangle the differential effects of SLEs on GMV change in MDD patients with recurrences during the two-year interval (T2-T1), we conducted exploratory whole-brain analyses comparing MDD patients with and without an episode and HCs. In a 3x2 repeated measures ANCOVA design in SPM, one significant cluster emerged in the left middle frontal, postcentral, and precentral gyrus (*k* = 3 736 voxels, x/y/z = -52/-26/44, *t*_1,743_ = 4.20 FWE cluster-level, Cohen’s *d* = 0.31, *p*<.001). HCs had larger GMV reductions in these areas the more SLEs they experienced during the two-year interval (middle frontal/postcentral/precentral gyri: *β*=-0.18, *t*=-3.45, *p*<.001). MDD patients with an episode had larger GMV increases in this area with increasing SLEs during the two-year interval (middle frontal/ postcentral/precentral gyri: *β*=0.22, *t*=2.83, *p*=.005). MDD patients without an episode showed no such GMV changes (middle frontal/postcentral/precentral gyri: *β*=-0.01, *t*=-0.16, *p*=.873). The identified cluster is located within the Dartel space Neuromorphometrics atlas and lies in 57% in the middle frontal gyrus, 16% in the postcentral gyrus, and 12% in the precentral gyrus.

| **Figure S1.** Association between stressful life events (LEQ) and GMV change between MDD patients with and without an episode and HCs during the two-year interval (T2-T1) |
| --- |
| Middle frontal / precentral / postcentral gyri |
| **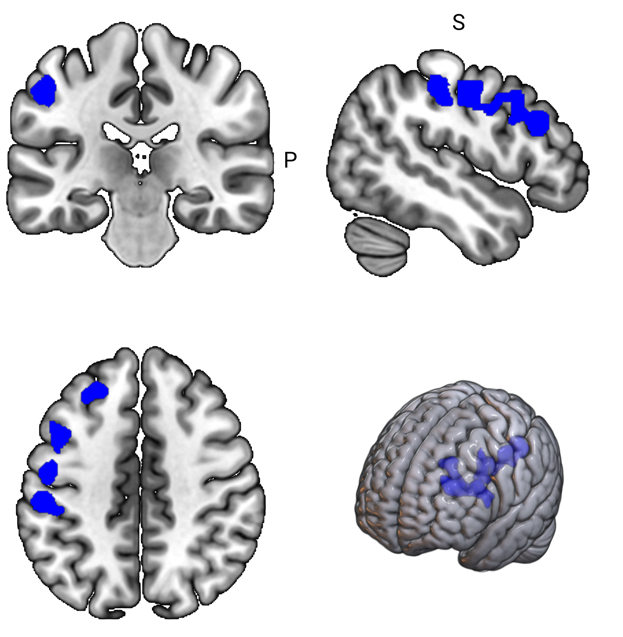**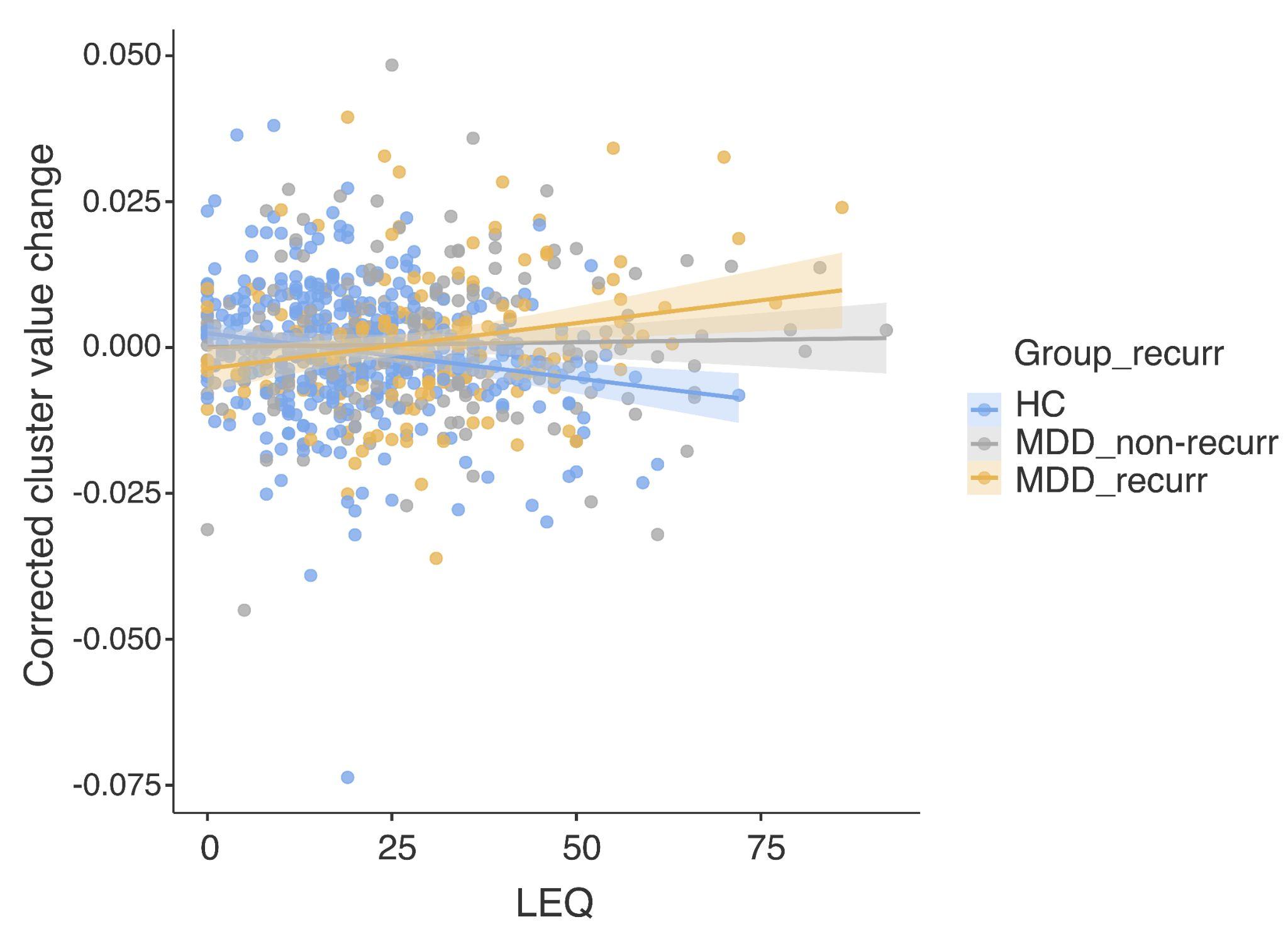 |
|  |

| **Table S6:** Model coefficients of three-way interactions between stressful life events (LEQ), childhood maltreatment (CTQ), and at least one depressive episode (yes vs. no vs. HC) on GMV changes (T2-T1) in the left middle frontal / precentral / postcentral gyri (LEQ x CTQ x recurrence group) | | | | | |
| --- | --- | --- | --- | --- | --- |
| **Predictor** | ***β*** | ***B*** | **SE** | ***t*** | ***p*** |
| **Left middle frontal/precentral/postcentral gyri** |  |  |  |  |  |
| Intercept |  | -0.02 | 0.01 | -2.38 | .018 |
| Interscan interval | -0.02 | -0.00 | 0.00 | -0.47 | .639 |
| Body-coil change | -0.05 | -0.00 | 0.00 | -1.46 | .145 |
| Gradient-coil change | -0.10 | -0.00 | 0.00 | -2.63 | .009 |
| Age | 0.08 | 0.00 | 0.00 | 2.03 | .043 |
| Sex | 0.09 | 0.00 | 0.00 | 2.50 | .013 |
| LEQ | -0.08 | 0.00 | 0.00 | 1.24 | .214 |
| CTQ | -0.05 | 0.00 | 0.00 | 2.37 | .018 |
| Recurrence group | 0.07 | 0.01 | 0.00 | 1.77 | .078 |
| LEQ ✻ CTQ | -0.06 | -0.00 | 0.00 | -2.59 | .010 |
| LEQ ✻ Recurrence group | 0.18 | -0.00 | 0.00 | -1.09 | .278 |
| CTQ ✻ Recurrence group | -0.06 | -0.00 | 0.00 | -2.72 | .007 |
| LEQ ✻ CTQ ✻ Recurrence group | 0.11 | 0.00 | 0.00 | 2.67 | .008 |
| *Note.* Middle frontal/precentral/postcentral gyri*: F*(12,722)=4.56, *p*<.001. | | | | | |

**Supplementary Results 3. Moderation of hsCRP on the association between SLEs and GMV change**

To explore potential biological indicators influencing the effects of SLEs on GMV change during the two-year interval (T2-T1), we ran exploratory moderation analyses using baseline (T1) hsCRP on the extracted means of intensity values of the identified clusters.

**Exploratory linear regression analyses.** We found a significant three-way interaction between LEQ total events score, hsCRP, and recurrence group on GMV change in the middle frontal/precentral/postcentral gyri (*β*=0.11, *t*=2.03, *p*=.043; for model coefficients, see Supplementary Table S7). Specifically, among MDD patients who had an episode during the two-year interval, higher levels of SLEs were related to GMV increases in the middle frontal, precentral, and postcentral gyri in the context of elevated baseline (T1) hsCRP levels (*β*=0.37, *t*=2.86, *p*=.005; see Figure S2), as compared to those patients without an episode (*β*=0.04, *t*=0.52, *p*=.601) or HCs (*β*=0.00, *t*=0.01, *p*=.990). This three-way association remained consistent without using any cut-off value for hsCRP (*β*=0.12, *t*=2.10, *p*=.036), as has been previously proposed [15]. No significant three-way interaction was observed between the LEQ total events score, hsCRP at baseline (T1), and group (MDD vs. HC) on GMV changes in both clusters (middle frontal gyrus: *β*=0.04, *t*=0.71, *p*=.479; precentral/postcentral gyri: *β*=0.02, *t*=0.38, *p*=.701; see Supplementary Table S8). Detailed descriptive statistics of the recurrence groups are provided in Supplementary Table S9.

| **Figure S2.** Three-way interaction between stressful life events (LEQ) and baseline (T1) high-sensitivity C-reactive protein (hsCRP) on GMV change in the middle frontal / precentral / postcentral gyri between MDD patients with and without an episode during the two-year interval and HCs | |
| --- | --- |
|  | MDD patients with episode* 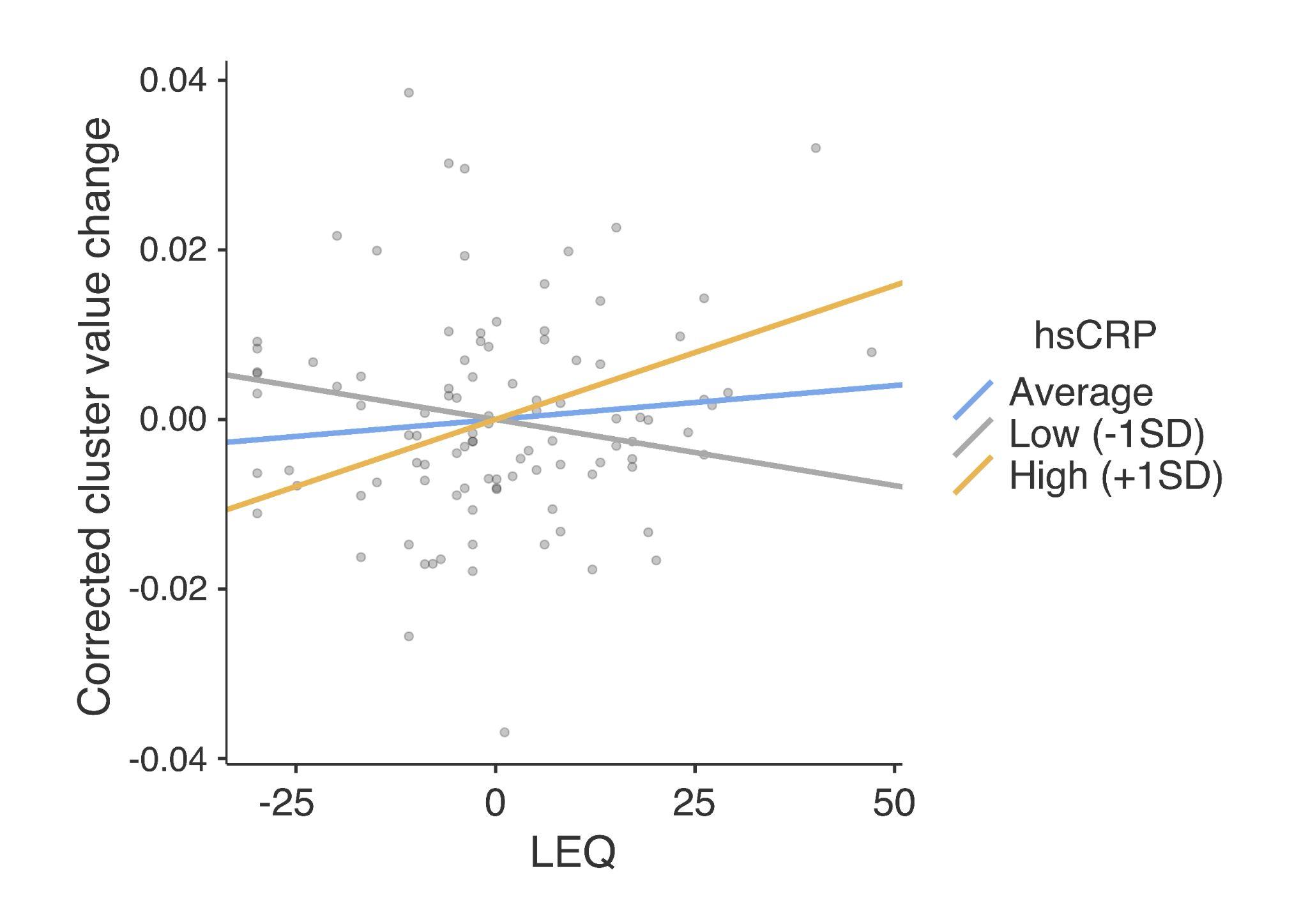 |
| *Note.* Figure S2 illustrates the moderating effect of hsCRP on the relationship between LEQ and GMV change in the middle frontal/precentral/postcentral gyri in MDD patients with at least one depressive episode. No significant moderating effect was observed for MDD patients without an episode or HCs during the two-year interval. The *β*_1_ value represents the simple effects of LEQ on GMV change, *β*_2_ value represents the two-way interaction between LEQ and hsCRP on GMV change, and *β*_3_ value represents the three-way interaction between LEQ, hsCRP, and the recurrence group including MDD patients with an episode, without an episode during the two-year interval and HCs on GMV change. *Interaction was statistically significant at *p*<.05. | |

| **Table S7:** Model coefficients of three-way interactions between stressful life events (LEQ), baseline (T1) high-sensitivity C-reactive protein (hsCRP), and at least one depressive episode (yes vs. no vs. HC) on GMV changes (T2-T1) in the left middle frontal / precentral / postcentral gyri (LEQ x hsCRP x recurrence group) | | | | | |
| --- | --- | --- | --- | --- | --- |
| **Predictor** | ***β*** | ***B*** | **SE** | ***t*** | ***p*** |
| **Middle frontal/precentral/postcentral gyri** |  |  |  |  |  |
| Intercept |  | -0.00 | 0.02 | -0.30 | .767 |
| Interscan interval | -0.02 | -0.00 | 0.00 | -0.43 | .668 |
| Body-coil change | -0.03 | -0.00 | 0.00 | -0.64 | .520 |
| Gradient-coil change | -0.10 | -0.00 | 0.00 | -2.16 | .031 |
| Age | 0.14 | 0.00 | 0.00 | 2.76 | .006 |
| Sex | 0.03 | 0.00 | 0.00 | 0.61 | .542 |
| BMI | -0.19 | -0.00 | 0.00 | -3.66 | <.001 |
| NSAID | -0.03 | -0.00 | 0.00 | -0.59 | .557 |
| Smoking status | -0.00 | -0.00 | 0.00 | -0.04 | .970 |
| Charge CRP | 0.03 | 0.00 | 0.00 | 0.77 | .443 |
| LEQ | -0.09 | -0.00 | 0.00 | -1.29 | .197 |
| hsCRP | 0.06 | 0.00 | 0.00 | 2.23 | .026 |
| Recurrence group | 0.09 | 0.00 | 0.00 | 0.56 | .577 |
| LEQ * hsCRP | 0.02 | -0.00 | 0.00 | -1.54 | .123 |
| LEQ * Recurrence group | 0.13 | 0.00 | 0.00 | 0.71 | .478 |
| hsCRP * Recurrence group | -0.06 | -0.00 | 0.00 | -2.31 | .021 |
| LEQ * hsCRP * Recurrence group | 0.11 | 0.00 | 0.00 | 2.03 | .043 |
| *Note.* Middle frontal/precentral/postcentral gyri*: F*(16,461)=2.92, *p*<.001. Cut-off score for baseline (T1) hsCRP were 3SD above the mean: 15.47 mg/l. The three-way association remained consistent without using any cut-off value for hsCRP (*β*=0.12, *t*=2.10, *p*=.036). | | | | | |

| **Table S8:** Model coefficients of three-way interactions between stressful life events (LEQ), baseline (T1) high-sensitivity C-reactive protein (hsCRP), and group (MDD vs. HC) on GMV changes (T2-T1) in the middle frontal / precentral / postcentral gyri (LEQ x hsCRP x group) | | | | | |
| --- | --- | --- | --- | --- | --- |
| **Predictor** | ***β*** | ***B*** | **SE** | ***t*** | ***p*** |
| **Left precentral/postcentral gyri** |  |  |  |  |  |
| Intercept |  | 0.01 | 0.02 | 0.31 | .758 |
| Interscan interval | -0.01 | -0.00 | 0.00 | -0.23 | .819 |
| Body-coil change | -0.04 | -0.00 | 0.00 | -0.89 | .374 |
| Gradient-coil change | -0.02 | -0.00 | 0.00 | -0.46 | .648 |
| Age | 0.08 | 0.00 | 0.00 | 1.63 | .103 |
| Sex | 0.06 | 0.00 | 0.00 | 1.31 | .191 |
| BMI | -0.20 | -0.00 | 0.00 | -3.78 | <.001 |
| NSAID | 0.00 | 0.00 | 0.00 | 0.07 | .942 |
| Smoking status | -0.02 | -0.00 | 0.00 | -0.33 | .740 |
| Charge CRP | 0.03 | 0.00 | 0.00 | 0.61 | .541 |
| LEQ | -0.16 | -0.00 | 0.00 | -2.98 | .003 |
| hsCRP | 0.06 | 0.00 | 0.00 | 0.85 | .393 |
| Group | 0.10 | -0.00 | 0.00 | -0.70 | .484 |
| LEQ * hsCRP | 0.05 | -0.00 | 0.00 | -0.05 | .963 |
| LEQ * Group | 0.16 | 0.00 | 0.00 | 2.36 | .019 |
| hsCRP * Group | -0.06 | -0.00 | 0.00 | -0.98 | .326 |
| LEQ * hsCRP * Group | 0.02 | 0.00 | 0.00 | 0.38 | .701 |
| **Left middle frontal gyrus** |  |  |  |  |  |
| Intercept |  | -0.01 | 0.02 | -0.37 | .713 |
| Interscan interval | -0.01 | -0.00 | 0.00 | -0.28 | .781 |
| Body-coil change | -0.01 | -0.00 | 0.00 | -0.28 | .777 |
| Gradient-coil change | -0.10 | -0.00 | 0.00 | -2.06 | .040 |
| Age | 0.17 | 0.00 | 0.00 | 3.48 | <.001 |
| Sex | 0.03 | 0.00 | 0.00 | 0.56 | .576 |
| BMI | -0.18 | -0.00 | 0.00 | -3.49 | <.001 |
| NSAID | -0.05 | -0.01 | 0.00 | -1.09 | .278 |
| Smoking status | -0.01 | -0.00 | 0.00 | -0.21 | .834 |
| Charge CRP | 0.04 | 0.00 | 0.00 | 0.85 | .394 |
| LEQ | -0.11 | -0.00 | 0.00 | -2.28 | .023 |
| hsCRP | 0.08 | 0.00 | 0.00 | 1.22 | .224 |
| Group | 0.12 | -0.00 | 0.00 | -0.20 | .842 |
| LEQ * hsCRP | 0.02 | -0.00 | 0.00 | -0.46 | .648 |
| LEQ * Group | 0.14 | 0.00 | 0.00 | 1.96 | .051 |
| hsCRP * Group | -0.05 | -0.00 | 0.00 | -1.16 | .245 |
| LEQ * hsCRP * Group | 0.04 | 0.00 | 0.00 | 0.71 | .479 |
| *Note.* Precentral/postcentral gyri: *F*(16,461)= 2.95, *p*<.001; middle frontal gyrus: *F*(16,461)=3.17, *p*<.001. | | | | | |

| **Table S9:** Descriptive statistics of HC, MDD non-recurrence, and MDD recurrence group at baseline (T1) and follow-up (T2) time points | | | | | | | | | |
| --- | --- | --- | --- | --- | --- | --- | --- | --- | --- |
|  | **Baseline (T1)** | | | | | **Follow-up (T2)** | | | |
|  | **HC**  (*n*=392) | **MDD non-recurr** (*n*=200) | **MDD recur**  (*n*=162) | ***P*** | **HC**  (*n*=392) | | **MDD non-recurr** (*n*=200) | **MDD recurr** (*n*=162) | ***P*** |
| Age | 34.97 (13.28) | 37.55 (13.30) | 32.90 (11.94) | .004 | 37.18 (13.27)** | | 39.84 (13.32)** | 35.13 (11.94)** | .003 |
| Sex, n | F = 238, M = 154 | F = 125, M = 75 | F = 105, M = 57 | .657 | - | | - | - | - |
| BMI | 24.21 (4.22) | 25.36 (5.16) | 26.01 (6.20) | .015 | 24.68 (4.10)** | | 26.19 (5.78)** | 26.86 (5.77)** | <.001 |
| TIV | 1533.63 (139.48) | 1537.06 (150.81) | 1518.35 (146.54) | .335 | 1531.18 (139.80)** | | 1534.72 (149.93)* | 1517.80 (146.24) | .396 |
| HAM-D | 1.24 (1.84) | 7.00 (5.83) | 9.39 (6.49) | <.001 | 1.05 (1.68)* | | 4.44 (5.16)** | 7.14 (5.43)** | <.001 |
| GAF | 91.65 (7.33) | 67.86 (14.33) | 62.96 (12.30) | <.001 | 90.40 (7.84)* | | 76.36 (13.29)** | 66.06 (12.56)* | <.001 |
| STAIS | 33.76 (8.09) | 47.06 (12.52) | 51.75 (11.39) | <.001 | 31.05 (7.84)* | | 38.42 (11.37)** | 45.46 (12.18)** | <.001 |
| FSozU/SSQ | 4.54 (0.50) | 3.94 (0.81) | 3.66 (0.88) | <.001 | 4.58 (0.48)* | | 4.14 (0.74)** | 3.87 (0.85)** | <.001 |
| PSS | 15.86 (6.99) | 27.38 (10.10) | 30.28 (8.62) | <.001 | 16.30 (6.61) | | 21.00 (8.18)** | 27.26 (8.56)** | <.001 |
| RS25 | 142.51 (17.48) | 116.94 (26.00) | 106.90 (22.41) | <.001 | 142.51 (16.57) | | 124.71 (21.77)** | 108.78 (22.23) | <.001 |
| RSQ secure, n (%) | 264 (67.34%) | 61 (30.50%) | 28 (17.28%) | <.001 | - | | - | - | - |
| NEOFFI neuroticism | 14.82 (7.20) | 26.10 (9.54) | 30.57 (8.43) | <.001 | - | | - | - | - |
| First-degree relative with MDD, BD, SCZ, or SZA, n (%) | 86 (21.93%) | 76 (38.00%) | 54 (33.33%) | <.001 | - | | - | - | - |
| hsCRP, mg/l | 1.95 (3.73) | 2.07 (3.59) | 3.33 (6.31) | .050^†^ | - | | - | - | - |
| Smoking status, n (%) | 36 (9.18%) | 44 (22%) | 29 (17.90%) | <.001 | - | | - | - | - |
| NSAID, n (%) | 0 (0%) | 7 (3.50%) | 2 (1.23%) | .001 | - | | - | - | - |
| Remission status | - | a = 74, r = 126 | a = 71, r = 90 | .172 | - | | a = 13, r = 187** | a = 41, r = 121** | <.001 |
| Antipsychotics, n (%) | - | 42 (21.00%) | 18 (11.11%) | .012 | - | | 18 (09.00%)* | 22 (13.58%) | .168 |
| Antidepressants, n (%) | - | 113 (56.50%) | 102 (62.96%) | .214 | - | | 77 (38.5%%)* | 83 (51.23%)* | .015 |
| Lithium, n (%) | - | 5 (2.50%) | 1 (0.01%) | .164 | - | | 7 (3.50%) | 4 (2.46%) | .570 |
| Number of reported SLEs between T1 and T2 | - | - | - | - | 11.06 (6.18) | | 13.91 (7.11) | 14.13 (6.95) | <.001 |
| LEQ total events score | - | - | - | - | 20.35 (13.64) | | 28.87 (17.98) | 29.96 (16.10) | <.001 |
| LEQ negative events score | - | - | - | - | 5.57 (6.11) | | 10.84 (10.57) | 13.90 (11.26) | <.001 |
| LEQ positive events score | - | - | - | - | 14.78 (10.45) | | 18.03 (13.27) | 16.07 (10.81) | .038 |
| CTQ | - | - | - | - | 31.51 (7.05) | | 41.97 (14.59) | 46.41 (15.28) | <.001 |
| Interscan interval, days | - | - | - | - | 808.00 (112) | | 814.30 (121.17) | 806.61 (110.49) | .329 |
| Number of depressive episodes between T1 and T2 | - | - | - | - | - | | - | 1.54 (0.84) | - |
| Duration of depressive episodes between T1 and T2 [months] | - | - | - | - | - | | 3.04 (6.85) | 7.17 (5.58) | <.001 |
| Number of hospitalizations between T1 and T2 | - | - | - | - | - | | 0.18 (0.58) | 0.49 (0.92) | <.001 |
| Duration of hospitalization between T1 and T2 [months] | - | - | - | - | - | | 0.80 (2.47) | 1.21 (2.86) | .104 |
| Note. All values are given as mean (SD) unless otherwise specified. BD, bipolar disorder; BMI, body mass index; CTQ, childhood trauma questionnaire; FSozU/SSQ, social support questionnaire; GAF, Global Assessment of Functioning; F, female, M, male; HAM-D, Hamilton Depression Rating Scale; HC, healthy control; hsCRP, high-sensitivity C-reactive protein; LEQ, Life Events Questionnaire; MDD, major depressive disorder; NEOFFI, NEO Five-Factor Inventory questionnaire; NSAID, nonsteroidal anti-inflammatory drugs; PSS, Perceived Stress Scale questionnaire; RSQ, Relationship Scales Questionnaire; RS25, 25-item Resilience Scale; SCZ, schizophrenia; SLEs, stressful life events; STAI-S, State-Trait Anxiety Inventory; SZA, schizoaffective disorder; a, acute; r, partially or fully remitted (according to SCID-I/DSM-IV-TR); n, number of participants. P-values stem from the non-parametric Kruskal-Wallis test for between-group comparisons or the Wilcoxon signed-rank test for within-group comparisons. *Significant within-group differences between baseline and follow-up at p<.05; **Significant within-group differences between baseline and follow-up at p<.001. ^†^hs-CRP data was only available for 509 participants (HCs: *n*=265 (67.6%); MDD non-recurr: *n*=136 (68%); MDD recurr: *n*=108 (66.6%)) | | | | | | | | | |

| **Table S10**: Three-way interactions between stressful life events (LEQ), childhood maltreatment, and clinical factors during the two-year interval on GMV change (T2-T1) in MDD patients (LEQ x CTQ x factor) | | |
| --- | --- | --- |
|  | | **Left middle frontal /**  **precentral / postcentral gyri** |
| **Duration of depressive episodes during interval** | *β* | -0.02 |
|  | *p* | .794 |
|  | *t* | -0.26 |
| **Number of depressive episodes during interval** | *β* | 0.21 |
|  | *p* | <.001* |
|  | *t* | 3.67 |
| **Duration of hospitalization during interval** | *β* | -0.02 |
|  | *p* | .816 |
|  | *t* | -0.23 |
| **Number of hospitalizations during interval** | *β* | -0.09 |
|  | *p* | .190 |
|  | *t* | -1.31 |
| **Remission status at follow-up** | *β* | 0.01 |
|  | *p* | .891 |
|  | *t* | 0.14 |
| *Note*. Using multiple linear regression, the interactions were assessed between the cluster intensity values and confounding variables that could have influenced the relationship between stressful life events and GMV change. Results indicate that stressful life events and childhood maltreatment did not interact with clinical variables other than depressive episodes on GMV change. *Results are significant after correction for multiple testing. | | |

| **Table S11**: Three-way interactions between stressful life events (LEQ) and clinical-psychosocial factors at follow-up (T2) on GMV changes (T2-T1) in MDD patients with and without an episode and HCs (LEQ x factors x recurrence group) | | |
| --- | --- | --- |
|  | | **Left middle frontal/**  **precentral/postcentral gyri** |
| **Family history of MDD/BD/SCZ/SZA** | *β* | 0.05 |
|  | *p* | .173 |
|  | *t* | 1.36 |
| **Remission status** | *β* | -0.04 |
|  | *p* | .476 |
|  | *t* | -0.71 |
| **Secure childhood Attachment** | *β* | -0.00 |
|  | *p* | .953 |
|  | *t* | -0.06 |
| **RS25** | *β* | -0.02 |
|  | *p* | .700 |
|  | *t* | -0.38 |
| **FSozU** | *β* | -0.02 |
|  | *p* | .681 |
|  | *t* | -0.41 |
| *Note*. BD, bipolar disorder; FSozU/SSQ, social support questionnaire; MDD, major depressive disorder; RS25, 25-item Resilience Scale, SZA, schizoaffective disorder; SCZ, schizophrenia. Using multiple linear regression, we assessed interactions between cluster intensity values and potential confounders of GMV change. Only childhood maltreatment, not familial risk, acute illness, attachment style, subjective resilience, or perceived social support interacted with stressful life events on GMV change in MDD patients with an episode during the interval. | | |

| **Figure S3.** Predictive association between cumulative stressful life events (LEQ total events score) during the two-year follow-up period (T2-T1) and baseline (T1) GMV in the precentral / postcentral gyri among groups |
| --- |
| Precentral / postcentral gyri |
| 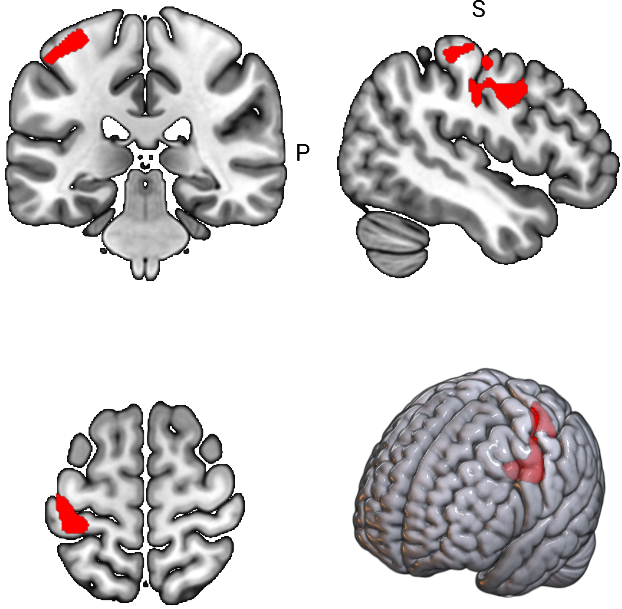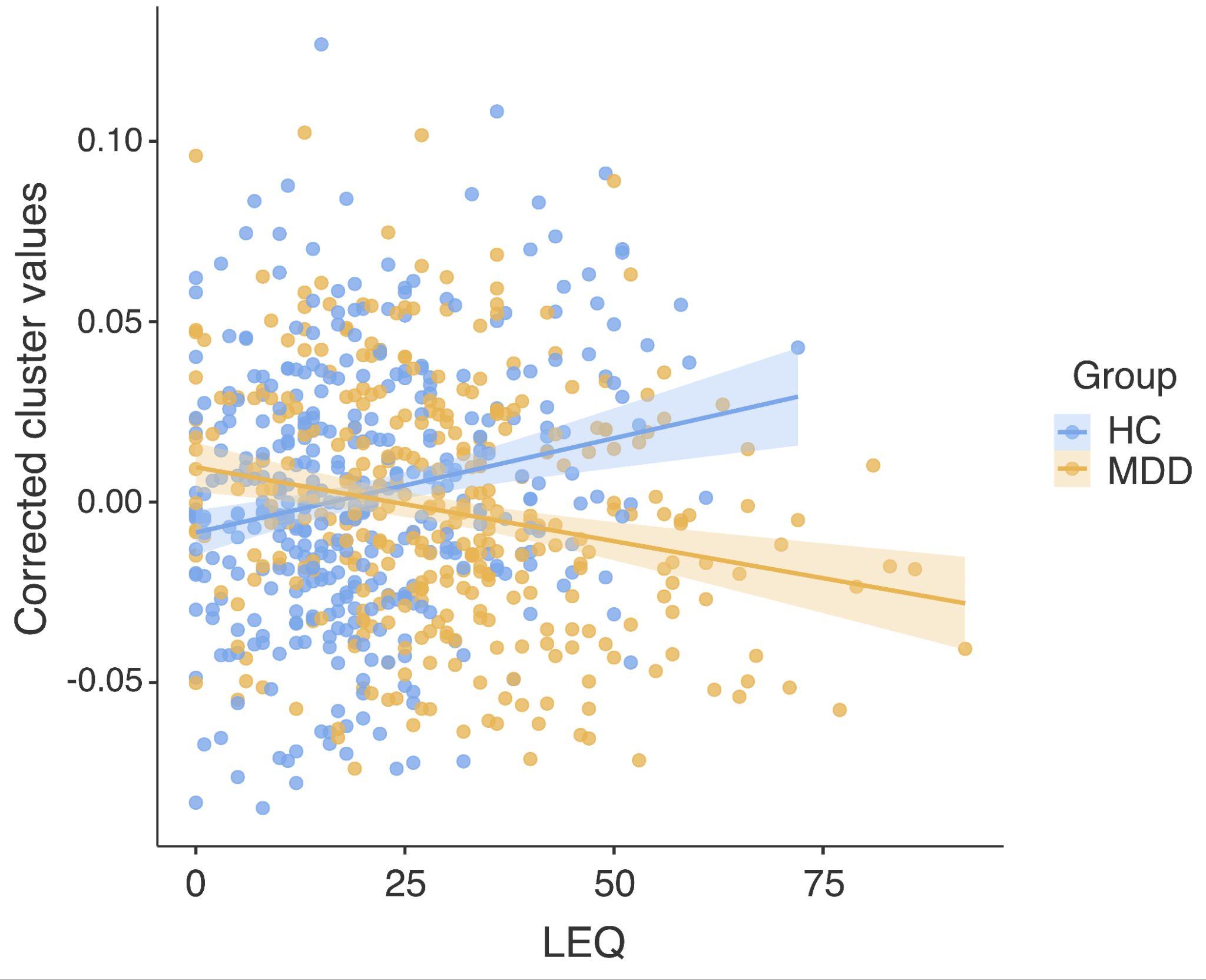 |
| *Note.* HCs showed increased GMV in the precentral and postcentral gyri at baseline (T1) time point, as the number of SLEs increased during the two-year interval (T2-T1), whereas MDD patients showed decreased GMV. |

| **Figure S4.** Cluster overlap of predictive baseline (T1; red) and longitudinal (T2-T1; blue) associations between stressful life events (LEQ) and GMV in the precentral / postcentral gyri |
| --- |
| Precentral / postcentral gyri |
| **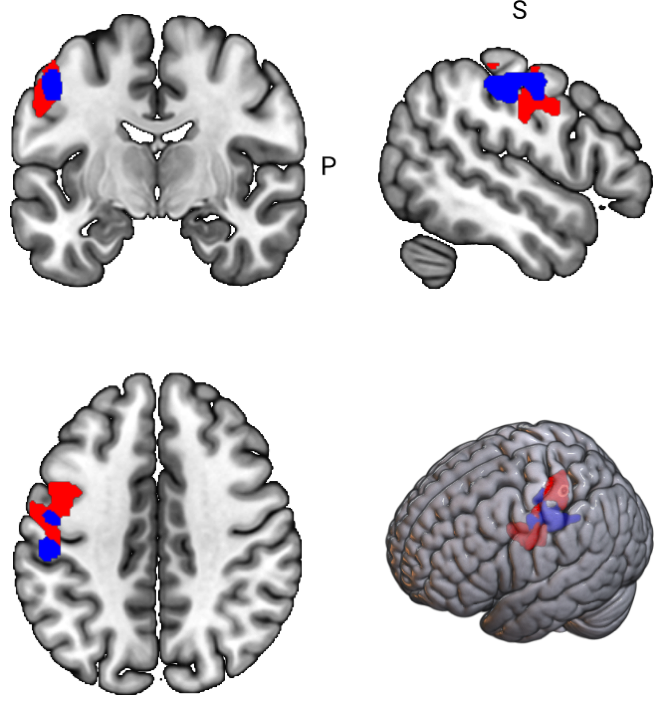** |
| *Note.* This finding suggests a significant overlap between predictive cross-sectional (T1) and longitudinal (T2-T1) clusters associated with stressful life events in the precentral and postcentral gyri. |

**References**

1. Quidé Y, Bortolasci CC, Spolding B, Kidnapillai S, Watkeys OJ, Cohen-Woods S, et al. Systemic inflammation and grey matter volume in schizophrenia and bipolar disorder: Moderation by childhood trauma severity. Prog Neuropsychopharmacol Biol Psychiatry. 2021;105:110013.

2. Taki Y, Thyreau B, Kinomura S, Sato K, Goto R, Wu K, et al. Correlation between high-sensitivity C-reactive protein and brain gray matter volume in healthy elderly subjects. Hum Brain Mapp. 2013;34:2418–2424.

3. Opel N, Cearns M, Clark S, Toben C, Grotegerd D, Heindel W, et al. Large-scale evidence for an association between low-grade peripheral inflammation and brain structural alterations in major depression in the BiDirect study. J Psychiatry Neurosci. 2019;44:423–431.

4. Mac Giollabhui N, Ng TH, Ellman LM, Alloy LB. The longitudinal associations of inflammatory biomarkers and depression revisited: systematic review, meta-analysis, and meta-regression. Mol Psychiatry. 2021;26:3302–3314.

5. Orsolini L, Pompili S, Tempia Valenta S, Salvi V, Volpe U. C-Reactive Protein as a Biomarker for Major Depressive Disorder? Int J Mol Sci. 2022;23:1616.

6. Johnson T V., Abbasi A, Master VA. Systematic Review of the Evidence of a Relationship Between Chronic Psychosocial Stress and C-Reactive Protein. Mol Diagn Ther. 2013;17:147–164.

7. Cathomas F, Holt LM, Parise EM, Liu J, Murrough JW, Casaccia P, et al. Beyond the neuron: Role of non-neuronal cells in stress disorders. Neuron. 2022;110:1116–1138.

8. Enache D, Pariante CM, Mondelli V. Markers of central inflammation in major depressive disorder: A systematic review and meta-analysis of studies examining cerebrospinal fluid, positron emission tomography and post-mortem brain tissue. Brain Behav Immun. 2019;81:24–40.

9. Gritti D, Delvecchio G, Ferro A, Bressi C, Brambilla P. Neuroinflammation in Major Depressive Disorder: A Review of PET Imaging Studies Examining the 18-kDa Translocator Protein. J Affect Disord. 2021;292:642–651.

10. Li H, Sagar AP, Kéri S. Translocator protein (18 kDa TSPO) binding, a marker of microglia, is reduced in major depression during cognitive-behavioral therapy. Prog Neuropsychopharmacol Biol Psychiatry. 2018;83:1–7.

11. Asan L, Falfán-Melgoza C, Beretta CA, Sack M, Zheng L, Weber-Fahr W, et al. Cellular correlates of gray matter volume changes in magnetic resonance morphometry identified by two-photon microscopy. Sci Rep. 2021;11:4234.

12. Green C, Shen X, Stevenson AJ, Conole ELS, Harris MA, Barbu MC, et al. Structural brain correlates of serum and epigenetic markers of inflammation in major depressive disorder. Brain Behav Immun. 2021;92:39–48.

13. Deng S, Chen J, Wang F. Microglia: A Central Player in Depression. Curr Med Sci. 2020;40:391–400.

14. Calcia MA, Bonsall DR, Bloomfield PS, Selvaraj S, Barichello T, Howes OD. Stress and neuroinflammation: a systematic review of the effects of stress on microglia and the implications for mental illness. Psychopharmacology (Berl). 2016;233:1637–1650.

15. Mac Giollabhui N, Ellman LM, Coe CL, Byrne ML, Abramson LY, Alloy LB. To exclude or not to exclude: Considerations and recommendations for C-reactive protein values higher than 10 mg/L. Brain Behav Immun. 2020;87:898–900.
